# Suicide rates and suicidal behaviour in displaced people: A systematic review

**DOI:** 10.1371/journal.pone.0263797

**Published:** 2022-03-10

**Authors:** Elise Cogo, Marylou Murray, Gemma Villanueva, Candyce Hamel, Paul Garner, Steven L. Senior, Nicholas Henschke

**Affiliations:** 1 Cochrane Response, London, United Kingdom; 2 Liverpool School of Tropical Medicine, Liverpool, United Kingdom; 3 University of Manchester, Manchester, United Kingdom; Washington University in St. Louis, UNITED STATES

## Abstract

**Background:**

Refugees, and other forcibly displaced people, face mental distress and may be disproportionately affected by risk factors for suicide. Little is known about suicidal behaviour in these highly mobile populations because collecting timely, relevant, and reliable data is challenging.

**Methods and findings:**

A systematic review was performed to identify studies of any design reporting on suicide, suicide attempts, or suicidal ideation among populations of displaced people. A sensitive electronic database search was performed in August 2020, and all retrieved studies were screened for relevance by two authors. Studies were categorised by the population being evaluated: refugees granted asylum, refugees living in temporary camps, asylum seekers, or internally displaced people. We distinguished between whether the sampling procedure in the studies was likely to be representative, or the sample examined a specific non-representative subgroup of displaced people (such as those already diagnosed with mental illness). Data on the rates of suicide or the prevalence of suicide attempts or suicidal ideation were extracted by one reviewer and verified by a second reviewer from each study and converted to common metrics. After screening 4347 articles, 87 reports of 77 unique studies were included. Of these, 53 were studies in representative samples, and 24 were based on samples of specific target populations. Most studies were conducted in high-income countries, and the most studied population subgroup was refugees granted asylum. There was substantial heterogeneity across data sources and measurement instruments utilised. Sample sizes of displaced people ranged from 33 to 196,941 in studies using general samples. Suicide rates varied considerably, from 4 to 290 per 100,000 person-years across studies. Only 8 studies were identified that compared suicide rates with the host population. The prevalence of suicide attempts ranged from 0.14% to 15.1% across all studies and varied according to the prevalence period evaluated. Suicidal ideation prevalence varied from 0.17% to 70.6% across studies. Among refugees granted asylum, there was evidence of a lower risk of suicide compared with the host population in 4 of 5 studies. In contrast, in asylum seekers there was evidence of a higher suicide risk in 2 of 3 studies, and of a higher risk of suicidal ideation among refugees living in camps in 2 of 3 studies compared to host populations.

**Conclusion:**

While multiple studies overall have been published in the literature on this topic, the evidence base is still sparse for refugees in camps, asylum seekers, and internally displaced people. Less than half of the included studies reported on suicide or suicide attempt outcomes, with most reporting on suicidal ideation. International research networks could usefully define criteria, definitions, and study designs to help standardise and facilitate more research in this important area.

**Registration:**

PROSPERO CRD42019137242.

## Introduction

Conflict remains a substantial threat to global population health. The number of people forcibly displaced is higher than ever with record levels of 82.4 million in 2020 (>1% of the world’s population), which is more than double compared to 10 years earlier [1]. Internally displaced people (IDP) make up the largest group of displaced people, at 48.0 million in 2020 [2]. The numbers of refugees and asylum seekers are estimated at 26.4 million and 4.1 million in 2020, respectively [2]. As conflicts become more protracted, the management of non-communicable disease and mental health have resulted in additional challenges [3, 4].

Suicide and suicide attempts have a profound impact on individuals, families, and communities. The World Health Organization (WHO) estimates that approximately 700,000 individuals died by suicide in 2019, with men dying at about twice the rates among women [5]. It is among the leading causes of death worldwide, with more deaths due to suicide than to malaria, breast cancer, war and homicide. The reduction of suicide mortality has been prioritized by the WHO as a global target and included as an indicator in the United Nations Sustainable Development Goals (SDGs) under target 3.4 [6, 7].

Refugees and other forcibly displaced people may be disproportionately affected by suicide risk [8, 9]. Mental health may be adversely impacted by displacement. Refugees face additional stressors during enforced departure from their homeland and on arrival in the host country such as discrimination, detention, language and cultural barriers [8]. ‘Losses’ identified include family and friends, homeland, status, community contact, language, financial assets, income, and financial security [10]. While most suicides are known to occur in low- and middle-income countries (79%) [11], the risks of suicide and suicidal behaviour among forcibly displaced people are unknown.

Previous reviews of suicide in refugee populations found a range of suicide proportions from 3.4% to 34% of recorded deaths [8]. Little is known about populations still in displacement because collecting timely, relevant, and reliable data is challenging in populations that are highly mobile. Suicide is also heavily stigmatized or even illegal in many countries, so suicidal behaviours may go under-reported and people at risk of suicide may be reluctant to seek help [12]. Regular monitoring of suicide across different risk groups is essential for effective national suicide prevention strategies [13]. This provides essential information for understanding the scope of the problem so that interventions can be tailored to meet the needs of specific populations.

This systematic review aims to synthesise what is known about the rates and prevalence of suicide and suicidal behaviour among displaced people from published literature.

## Methods

The protocol for this systematic review was registered with PROSPERO (CRD42019137242). In the review protocol it was planned to include studies of people who have been displaced because of natural disasters, but due to the large number of studies, estimates from these populations will now be reported in a separate systematic review. Initially, studies on populations affected by conflict but who have not been displaced were also considered for inclusion. However, the difficulty in defining these populations and the variable level of exposure to conflict, led the author team to decide against including these studies. Studies were also excluded if they reported prevalence estimates for returned asylum seekers or displaced populations (i.e. people not currently displaced), or for economic migrants.

### Inclusion criteria

#### Study designs

Studies of any design that could provide an estimate of the rate or prevalence of suicide or suicide attempts, or prevalence of suicidal ideation, in populations of displaced people were considered for inclusion. This included observational studies (longitudinal population-based cohort studies, case-control, and cross-sectional surveys) and baseline rates or prevalence in randomised controlled trials of interventions targeted at the populations of interest. Case reports, reviews, and case series were excluded. Publication status was not used to determine eligibility.

#### Populations

Studies were included if they reported on populations (of any age) of forcibly displaced people, which we defined as people who have had to leave their homes in the context of an emergency because of a deliberate event such as conflict or war. This includes those who identified as refugees or asylum seekers (i.e. displaced people who cross international borders) as well as internally displaced people (i.e. people who remain in their own country). A list of common definitions for terms used in this review are provided in Box 1.

**Box 1. Definitions of subgroups of displaced people used in this review.**

$$\begin{array}{cc} \text{Term} & \text{Definition}\\ \text{Refugees} & \text{People}\;\text{who}\;\text{have}\;\text{fled}\;\text{their}\;\text{country}\;\text{because}\;\text{they}\;\text{are}\;\text{at}\;\text{serious}\;\text{risk}\;\text{of}\;\text{human}\;\text{rights}\;\text{violations}\;\text{and}/\text{or}\;\text{persecution}.\;\text{They}\;\text{include}\;\text{individuals}\;\text{recognised}\;\text{under}\;\text{the}\;1951\;\text{Convention}\;\text{relating}\;\text{to}\;\text{the}\;\text{Status}\;\text{of}\;\text{Refugees};\;\text{its}\;1967\;\text{Protocol};\;\text{the}\;1969\;\text{OAU}\;\text{Convention}\;\text{Governing}\;\text{the}\;\text{Specific}\;\text{Aspects}\;\text{of}\;\text{Refugee}\;\text{Problems}\;\text{in}\;\text{Africa};\;\text{those}\;\text{recognised}\;\text{in}\;\text{accordance}\;\text{with}\;\text{the}\;\text{UNHCR}\;\text{Statute};\;\text{individuals}\;\text{granted}\;\text{complementary}\;\text{forms}\;\text{of}\;\text{protection};\;\text{or}\;\text{those}\;\text{enjoying}\;\text{temporary}\;\text{protection}.\;\text{Since}\;2007,\;\text{the}\;\text{refugee}\;\text{population}\;\text{also}\;\text{includes}\;\text{people}\;\text{in}\;\text{a}\;\text{refugee-like}\;\text{situation}.\\ \text{Internally}\;\text{displaced}\;\text{people} & \text{People}\;\text{or}\;\text{groups}\;\text{of}\;\text{individuals}\;\text{who}\;\text{have}\;\text{been}\;\text{forced}\;\text{to}\;\text{leave}\;\text{their}\;\text{homes}\;\text{or}\;\text{places}\;\text{of}\;\text{habitual}\;\text{residence},\;\text{in}\;\text{particular}\;\text{as}\;\text{a}\;\text{result}\;\text{of},\;\text{or}\;\text{in}\;\text{order}\;\text{to}\;\text{avoid}\;\text{the}\;\text{effects}\;\text{of}\;\text{armed}\;\text{conflict},\;\text{situations}\;\text{of}\;\text{generalised}\;\text{violence},\;\text{violations}\;\text{of}\;\text{human}\;\text{rights},\;\text{or}\;\text{natural}\;\text{disasters},\;\text{and}\;\text{who}\;\text{have}\;\text{not}\;\text{crossed}\;\text{an}\;\text{international}\;\text{border}.\\ \text{Asylum}\;\text{seekers} & \text{Individuals}\;\text{who}\;\text{have}\;\text{sought}\;\text{international}\;\text{protection}\;\text{and}\;\text{whose}\;\text{claims}\;\text{for}\;\text{refugee}\;\text{status}\;\text{have}\;\text{not}\;\text{yet}\;\text{been}\;\text{determined},\;\text{irrespective}\;\text{of}\;\text{when}\;\text{they}\;\text{may}\;\text{have}\;\text{been}\;\text{lodged}. \end{array}$$

#### Outcomes

All studies reporting data on rate or prevalence of suicide, suicide attempts, and suicidal ideation among populations of displaced people were considered for inclusion. For this review we consider suicidal ideation to mean having thoughts or ideas about the possibility of ending one’s life, a suicide attempt to be an attempt to die by suicide that results in survival, and suicide to be intentionally causing one’s death. These outcomes were defined in each study by the authors of the primary studies. We considered suicide estimates measured by death registers, surveillance systems, autopsies (verbal and recorded), and medical records, and estimates of suicidal attempts and ideation measured using validated tools or survey questions.

As the design of the included studies varied, the source and type of data reported for these outcomes also varied. All relevant data for these outcomes was extracted and presented according to data source, host country, and prevalence period (i.e. lifetime prevalence, point prevalence). Where available, data were also extracted on comparative estimates between displaced people and host or general populations.

#### Search methods

A broad search strategy informed by experts in this area was developed to maximise sensitivity, combining medical subject headings and free text terms (see S1 Table) to identify relevant studies in the following databases:

- MEDLINE (Pubmed)
- Embase (OVID)
- CINAHL (Cumulative Index to Nursing and Allied Health Literature, EBSCOHost)
- PsycINFO (EBSCOHost)
- Social Science Citation Index (SSCI, Web of Science)
- Scopus (Elsevier)
- Global Index Medicus at http://search.bvsalud.org/ghl/index.php
- Suicide Information and Education Collection (SIEC) database

Searches were conducted in April 2019 and updated in August 2020. Searches were not limited by publication date, geographical location, or language. The reference lists of relevant articles and reviews were screened to identify additional studies.

#### Selection of studies

Two reviewers independently screened the abstracts of all studies identified by the search strategy. Following this stage, two reviewers also independently screened full texts of studies appearing to meet the inclusion criteria. Discrepancies were discussed among the reviewers, and where necessary a third reviewer assessed the study under discussion until a consensus was reached.

#### Data extraction and management

One reviewer extracted study characteristics and relevant outcome data from all included studies. A second reviewer cross-checked all extracted data and any discrepancies between the two reviewers were discussed. Relevant information extracted from each study included: population characteristics (such as ethnicity, age, religion, trauma exposure, and duration of resettlement), setting, study design, study dates, data collection method and source of data. For each of the three outcomes of interest, the number of participants analysed as well as summary statistics were extracted where available. Host countries/territories (where studies were conducted) were classified into the four World Bank income levels (i.e., low, lower middle, upper middle, high).

#### Methodological quality

The search strategy was designed to capture the breadth of the available literature in this population. As a result, a variety of study designs were considered for inclusion in this review. This prevented the use of a single checklist approach to assessing methodological quality and required a more pragmatic approach. The assessment in this review was based on the study design and the source from which data were obtained. Using this pragmatic approach, we judged studies drawing on vital events registration, specific demographic surveillance, and longitudinal population-based cohorts to be of higher quality while studies drawing on data from retrospective cohorts/analysis and cross-sectional surveys were considered low quality.

#### Data synthesis

The following common metrics were calculated using the extracted data for each study, when possible, to facilitate comparison across studies, population groups, and settings: suicide rate per 100,000 person-years, prevalence of suicide attempt in percentage, and prevalence of suicidal ideation in percentage with 95% confidence intervals (CIs). Where data were also available comparing the rates or prevalence of the three outcomes in displaced people with the general population of the host country, relative estimates (relative risks or hazard ratios) with 95% CIs were calculated and presented.

We chose not to pool rates of suicide across studies due to the heterogeneity of study designs and used narrative syntheses with tabulated and textual descriptions systematically reporting results for the three aspects of suicidal behaviour. Comparative results with host populations were synthesised using the vote counting based on the direction of effect method [14, 15].

The reporting of studies and results in this review are grouped into two main categories, based on their likely representativeness to the target population or to a target subgroup (for example, adolescents, pregnant women):

1. studies using *representative* or *non-selected* samples of the target group (that is, evaluating where possible the whole population or whole subgroup, as applicable), referred to below as “general samples”, and
2. studies using *selected samples* of the target group based on their inclusion criteria that may confound the outcomes (for example, known high risk groups within the populations of displaced people, such as those already diagnosed with mental illness), referred to below as “specific samples”.

Furthermore, within each category results are presented separately for the following five population subgroups:

1. refugees granted permanent asylum status,
2. refugees living in refugee camps or with only temporary protection,
3. asylum seekers,
4. mixed samples of refugees & asylum seekers, and
5. internally displaced people.

Due to possible overlap, if a study reports the population as asylum seekers living in camps, this was categorised under refugees living in camps.

## Results

### Included studies

**Fig 1:**
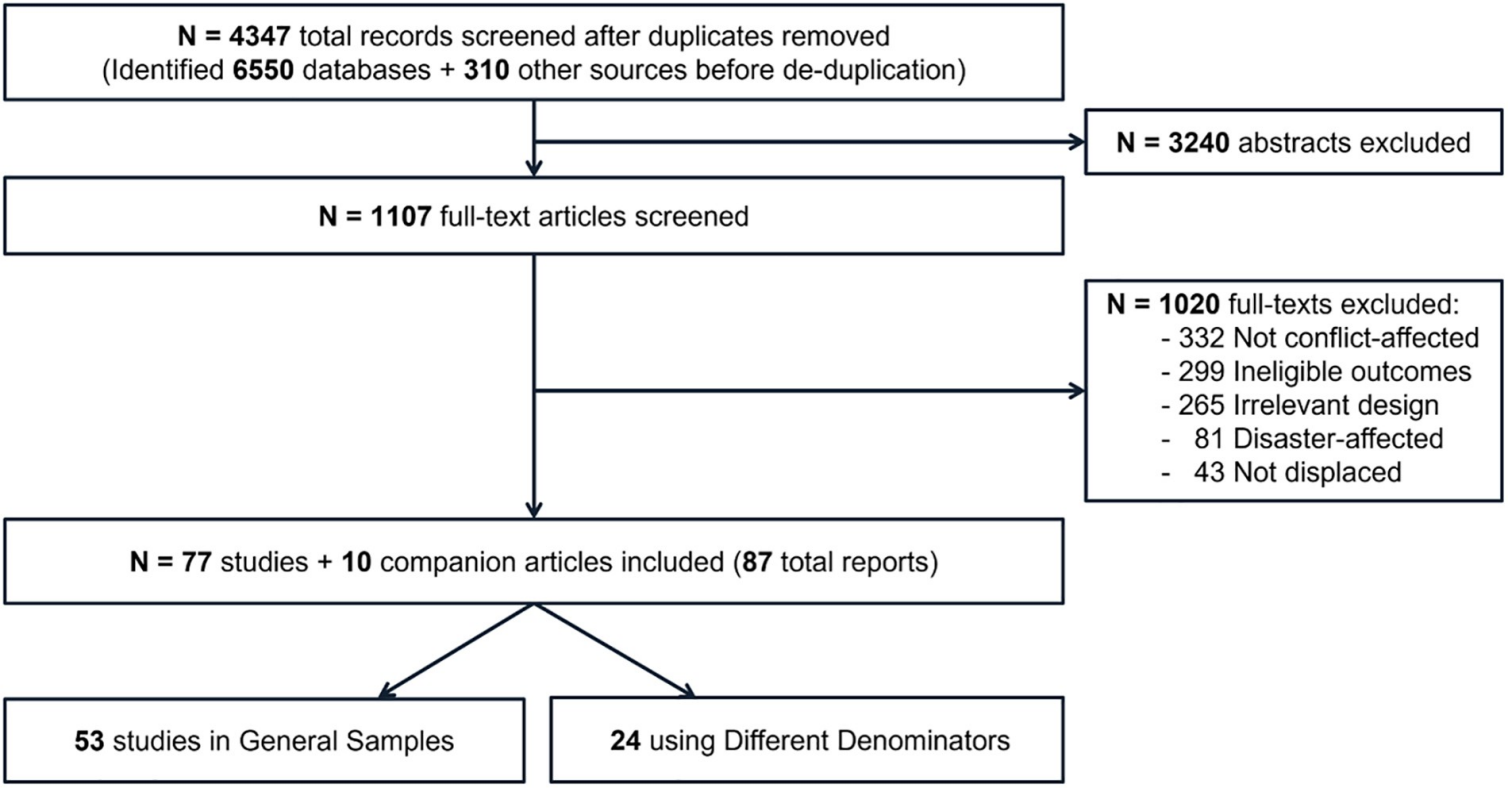
PRISMA flow diagram.

A total of 6899 records were identified by the electronic searches. Following removal of duplicates, 4347 abstracts were screened by two reviewers. Of these, 1107 full-text articles were considered potentially relevant. Finally, 87 articles reporting on 77 studies [16–92] plus 10 companion articles [93–102] were included in this review. Reasons for study exclusions are outlined in the PRISMA flow diagram (Fig 1). Five of the included articles were published in languages other than English. These were Danish, Dutch, two in German, and Spanish [50, 79, 83, 90, 99].

Of the 77 included studies, 53 were in general samples and 24 used specific samples. The types of data sources used for each outcome are presented below. Characteristics of the 53 included studies and their populations that used general samples are presented in Table 1, grouped by population subgroup. Additional characteristics are also reported in the corresponding outcome results tables. Study characteristics and results for the specific samples (n = 24 studies) are reported in S2 Table. The various samples evaluated by these studies included people diagnosed/referred for mental health conditions, intoxication-related emergency department admissions, forensic autopsies ordered by attorney’s office, and emergency department admissions that required a forensic exam.

**Table 1:** Characteristics of included studies using general samples, grouped by population type.

Author, Pub. Year	Population Summary	Setting	Ethnicity (Region of Origin)	Age* (y)	Gender	Religion	Trauma Exposure	Duration of Resettlement* (y)	Data Collection
									Source or Measurement
***Refugees granted permanent asylum status***:									
Alley 1982 [18]	Indochinese refugees who had entered Utah (USA) beginning in 1978	Community	All Indochinese	NR	NR	NR	NR	NR	Survey
Amin 2021 [20]	9-year cohort aged 16–64 years on Dec 31, 2004	Community	Eritrean, Ethiopian, Somali, Afghani, Iranian, Iraqi, Syrian, Chilean, former Yugoslavian	range: 16–64	M- 58%	NR	NR	NR	National Cause of Death and inpatient records registers
Ao 2016 [21]	Adults in 7 cities in Georgia, New York, Arizona, and Texas	Community	All Bhutanese	median 34 (range: 18–83)	Mixed	72% Hindu, 10% Buddhist, 16% Christian	36% had experienced 4–7 traumatic events pre-migration, and 34% had experienced ≥8	median: 1.8	Beck Scale for Suicidal Ideation
								(Range: 0.2–5)	
Bhui 2003 [25]	Somali refugees in the London Borough of Greenwich (UK)	Community	All Somali	mean: 40.4; range: 20–88	M- 51%	NR	Shortage of food and water, no shelter, poor health, torture, imprisonment, murder, separated from family and enforced isolation	Men: 7.9; Women: 8.2	BDI Suicide items
Choi 2020 [30]	Youth in 7^th^-12^th^ grade with either father/ mother born in North Korea	Classrooms in middle and high schools	All North Korean	NR	F- 41%	NR	NR	NR	Korean Youth’s Risk Behavior Survey
Hollander 2020 [42]	Cohort aged 16 and up who were born between 1975 and 1984	Community	19% former Yugoslavian, 18% Iraqi, 13% Central Asian, 11% Somali or Eritrean or Ethiopian or Djibouti, 11% Southeast Asian, 10% Iranian, 7% Baltics or Russian, 7% North Asian, 4% North African	24.8 (7.0)	M- 59%	NR	NR	1–6	Causes of Death Register
								(at cohort entry)	
Jankovic 2013 [47]	Refugees from former Yugoslavia who experienced war trauma residing in UK, Italy, Germany	NR	All from former Yugoslavia	41.6	M- 49%	NR	Mean war events: 6.8	NR	MINI International Neuropsychiatric Interview
				(10.8)					
Meyerhoff 2020 [55]	Adults living in the greater Burlington, Vermont region	a local community center and a community mental health clinic serving refugees	All Bhutanese	mean: 41	M- 52%	65% Hindu, 17% Buddhist, 13% Christian, 5% Kirat	NR	NR	Beck Scale for Suicidal Ideation
Nickerson 2019 [60]	Adults literate in Arabic, Farsi, Tamil or English	Community	67% Iraqi, 20% Syrian, 6% Iranian, 3% Afghani	39.0 (12.5)	M- 53%	NR	potentially traumatic events: mean 3.30 (SD 3.30)	1.44 (1.37)	PHQ-9 (item 9) questionnaire and additional suicidal intent questions
Noh 2017 [61]	North Korean refugees living in Seoul (South Korea)	Hana Center (a representative welfare center supported by the Korean government to assist NKRs in South Korea)	All North Korean	≥30 years	M- 23%	NR	NR	68% been in South Korea for <5	Health questionnaire
Norredam 2013 [62]	Refugees in Denmark from January 1993 to December 1999 and native Danes	Community	57% former Yugoslavian, 16% North African, 11% Iraqi, 10% other Middle Eastern	>18 years	Males: Refugees 56%; Danish-born 56%	NR	NR	NR	Danish Register on Causes of Death
Park 2018 [64]	North Korean refugee youth living in South Korea	Schools	Born in North Korea or China	mean 19; range: 13 to 17	M- 33%	NR	NR	NR	Questionnaire
Salama 2020^†^ [71]	Migrants living in 6 big cities	Community	59% Kurdish, 41% Somali	range: 18–64	F- 52%	Somalis and the majority of Kurds (75%) were Muslim	NR	>1	Hopkins Symptoms Checklist-25 (suicidal ideation item of HSCL-25)
Saunders 2017 [72]	Youth living in Ontario	Community	Mixed	range: 10–24	M-~50%	NR	NR	Range: 0–10	Ontario Registrar General Vital Statistics-Deaths registry
Saunders 2019 [73]	Adults living in Ontario for <10 years	Community	Mixed	NR	Mixed	NR	NR	<10	National Ambulatory Care Reporting System and Ontario Registrar General Vital Statistics-Deaths registries
Sobhanian 2006 [76]	Former refugee detainees from the Woomera Detention Centre living Australia	Community	Iranian and Afghani	31.8 (9.2); range: 18 to 70	M- 69%	NR	NR	Time in the detention centre ranged from 2 to 21 months; 11.30 months (3.80); Time living in the community ranged from 1 to 20; mean 6.97 months	The Truncated Assessment of Self-Destructive
									Thoughts (TASDT)
Tousignant1999 [85]	Adolescents from refugee families living in Canada	Schools	35 countries of origin were represented: El Salvador, Cambodia, Laos, Iran, Vietnam and the regions of Southeast Asia and Central America	mean: 15.7; range: 13 to 19	M- 48%	NR	NR	NR	Diagnostic Interview Schedule for Children Version 2.25 (DISC-2.25) for DSM-III-R diagnoses
Um 2020 [86]	Adults living in Seoul, Gyeonggi Province, and Incheon	Household	All North Korean	mean: 40.6	F- 67%	68% Christian	NR	mean: 6.3	5-item suicidal ideation scale
Vigod 2019 [87]	Mothers within 1 year postpartum in Ontario	Community	Mixed	mean: 30	F- 100%	NR	NR	NR	National Ambulatory Care Reporting System database
Westman 2006 [89]	National cohort aged 25–64 years	Community	Eastern European, Middle Eastern, other non-European countries^‡^	range: 25–64	Mixed (disaggregated below)	NR	NR	NR	National Cause of Death Register
***Refugees in refugee camps or with temporary protection***:									
Akinyemi 2012 [16]	Adults residing >1 year in Oru Refugee Camp	Refugee camp	66% Liberian, 33% Sierra-Leonean, 1% Togolese	34.8 (12.8)	M- 59%	70% Christian, 30% Muslim	NR	8.6 (4.8)	Mini-International Neuropsychiatric Interview (MINI)
Al-Modallal 2012 [17]	Refugee women attending healthcare centers in the cities of Amman, Zarqa, and Irbid (Jordan)	Health care centers in refugee camps	NR	Range: 16 to 62	F- 100%	NR	NR	NR	Survey
Falb 2013 [32]	Refugee women living in 3 camps along the Thai-Burma Border	Refugee camps	Mostly Burmese (79% Karen, 21% other)	32.1 (8.4)	F- 100%	Christian: 54%; other 46%	80/848 reported conflict victimization	NR	Questionnaire
Fellmeth 2016 [33]	Migrant and refugee pregnant women in a refugee camp along the Thailand-Myanmar border	Refugee camp	Mostly Burmese	Women who died by suicide: 18 to 36	F- 100%	NR	NR	NR	Clinic review of maternal deaths (MMR among live births)
Hermans 2017 [39]	Refugees in long-stay refugee camps at Lesbos (Greece)	Refugee camps	36% Syrian, 28% Afghani, 20% Pakistani	Median: 23 (IQR 28–38); 23% children	NR	NR	NR	NR	Clinical assessment
Itani 2017a [45]	Middle school students in Gaza and West Bank	Occupied Palestinian Territory and UNRWA refugee camps’ schools	All Palestinian	most 13–15	F- 53%	NR	NR	NR	Global School-based Student Health Survey
Itani 2017b [45]	Middle school students in Jordan	UNRWA refugee camp schools	All Palestinian	most 13–15	M- 52%	NR	NR	NR	Global School-based Student Health Survey
Itani 2017c [45]	Middle school students in Lebanon	UNRWA refugee camp schools	All Palestinian	most 13–15	F- 54%	NR	NR	NR	Global School-based Student Health Survey
Itani 2017d [45]	Middle school students in Syria	UNRWA refugee camp schools	All Palestinian	most 13–15	M- 51%	NR	NR	NR	Global School-based Student Health Survey
Rahman 2003 [66]	Afghan mothers in refugee camps in Pakistan	Refugee camps	All Afghani	28.2 (7.3)	F- 100%	NR	NR	13 (3.4) months	Pushto version of the Self-Reporting Questionnaire (SRQ- 20)
Slodnjak 2002 [75]	Refugee students from Bosnia and Herzegovina and Slovenian students from same age and school grade	School	All Bosnian and Herzegovina	14 to 15	M—refugees: 47%; Slovenian-born: 49%	NR	80% of the student refugees and 90% of their relatives had experienced war events	NR	Interviews
Ssenyonga 2012 [78]	Adults resident at Nakivale camp	Refugee camp	All Congolese (DRC)	≥18	Mixed	NR	Domestic violence exposure: mean 11.3 (SD 6.3) lifetime events, and 2.1 (SD 3.6) ongoing events	NR	Survey
Vijayakumar 2017 [88]	Residents of refugee camps in Tamil Nadu, South India	2 refugee camps	All Sri Lankan	mean: 40	F- 58%	71% Hindu, 29% Christian, 1% Muslim	Trauma during war: a) 31% lost/got separated from significant family member/friend; b) 27% confirmed/presumed death of family member/friend; c) 16% lost/got separated from 1st-degree relative	mean: 18.3	Beck’s Scale for Suicidal Ideation, and additional questions
Yu 2008 [91]	Refugees in protective facilities under the South Korean government protection in 2 Chinese cities	Protective facilities	All North Korean	range: 15–59	F- 71%	NR	NR	range: from under 1 to over 5	Personality Assessment Index (Suicide Ideation subscale of PAI; Morey 1991)
***Mixed refugees populations***:									
Stein 2010 [81]	Refugees sub-group among general populations surveyed in 21 low-, middle-, and high-income countries	NR	Nigeria, South Africa, Brazil, Colombia, Mexico, United States, India, Japan, New Zealand, China, Belgium, Bulgaria, France, Germany, Italy, the Netherlands, Romania, Spain, Ukraine, Israel, Lebanon	>18	Mixed	NR	NR	NR	World Mental Health (WMH) version of the WHO Composite International Diagnostic Interview (CIDI) and Suicidality Module of the WMH-CIDI
***Asylum seekers***:									
Cheney 2017 [29]	Male-to-female transgender asylum seekers in California	An advocacy and immigration legal services organization	All Mexican	mean: 32 (range: 20–58)	100% male-to-female transgender	NR	All had experienced physical and/or sexual assaults	NR	Review of declaration documents that were part of asylum application, including professional psychological evaluation
Cohen 2008 [31]	Asylum seekers in immigration removal centres and prisons	Immigration removal centres and prisons	Mixed	NR	Mixed	NR	NR	NR	Specific monitoring data (from medical records)
Fuhrer 2016 [35]	Asylum seekers in Halle (Germany)	NR	Mixed	>15 years	M- 85%	NR	NR	NR	Hopkins-Symptom-Checklist 25 (HSCL 25) and the Harvard Trauma Questionnaire
			(Had to speak Arab, Farsi/Dari, French, Hindi or English)						
Goosen 2011 [37]	Asylum seekers living in reception centres	Residential asylum seeker reception centres	Afghani, Iraqi, Angolan, Congolese (DRC), Azerbaijani, former Yugoslavian, Somali, Sudanese	≥15	Mixed (disaggregated below)	NR	NR	NR	A national standard suicidal behaviour notification form
Grupp 2020 [38]	Adults fluent in English, German, French, Farsi, Arabic, Kurdish, or Tigrinya	in their accommodation, at meeting points for asylum seekers, or through language courses	28% Iranian, 19% Eritrean, 17% Somali, 10% Afghani, 8% Cameroonian, 7% Syrian	30.5 (8.1)	M- 66%	NR	NR	15.1 months	PHQ-9
								(23.2)	
Huemer 2011 [44]	Unaccompanied minors aged 15–18 years	8 residential accommodations for unaccompanied refugee minors	34% Gambian, 27% Somali, 20% Nigerian, 5% Guinea-Bissau, 14% other African	17.0 (0.8)	M- 85%	66% Muslim, 22% Christian	NR	NR	Mini-International Neuropsychiatric Interview for children and adolescents (MINI Kid)
Keller 2003 [48]	Detained asylum seekers in New York, New Jersey, and Pennsylvania	Detention centers	77% African, 10% Eastern European, 6% Asian, 4% South American, 3% Middle Eastern	28 (7.3)	M- 80%	NR	74% had been tortured before immigration, 59% reported murder of family member or friend, 26% reported sexual assault	NR	Self-report in physician interview
Koppenaal 2003 [50]	Asylum seekers accommodated in housing facilities of the Central Organ Asylum Seekers	Housing facilities for asylum seekers	Mixed	All ages included	Mixed	NR	NR	NR	Statistics Netherlands causes of death list
Mittendorfer-Rutz 2020 [57]	Unaccompanied minors/ youth	4 state agencies and 2 NGOs	Mixed	range: 10–21	Mixed	NR	NR	NR	NGOs data and reports to the Health and Social Care Inspectorate. Validated using the national forensic autopsies register database
Nickerson 2019 [60]	Adults literate in Arabic, Farsi, Tamil or English	Community	48% Iranian, 22% Sri Lankan, 12% Iraqi, 4% Afghani	35.4 (8.6)	M- 69%	NR	PTE: mean 7.21 (SD 4.83)	3.65 (1.32)	PHQ-9 (item 9) questionnaire, and additional suicidal intent question
Staehr 2006 [79]	Asylum seekers aged 15 and up	Hospitals and Red Cross asylum centres	20% Iraqi, 15% Bosnian-Hercegovina, 14% Afghani, 6% Kosovon, 4% Iranian, 41% Other	≥15	Mixed	NR	~1/3 of those with suicidal behaviour experienced imprisonment and torture/war, 4 reported rape, and 4 forced marriage	mean: 12.3 months	Asylum centre reports and hospital records
Steel 2004 [80]	10 families from one ethnic group held for a protracted period	A remote detention facility	NR (all same ethnic group; 14 parents + their 20 children)	Ranges: parents 28–44; children 3–19	F-64% (parents)	71% Muslim	All adults reported traumatic experiences in their country of origin, with it being common for one parent to have been imprisoned and tortured for political reasons. All families reported fleeing their country of origin out of fear for the life of one or all of the family members. All families described traumatic experiences in detention.	mean: 2.3 (range: 2.0–2.7)	Structured Clinical Interview for DSM-IV Axis I Disorders; and Schedule for Affective Disorders and Schizophrenia for School-Age Children
Sultan 2001 [82]	Detained refugees in Sydney	Detention centre	Afghani, Iraqi, Iranian, former Yugoslavian, etc.	NR	M- 85%	NR	58% had been physically tortured, 27% experienced the murder or disappearance of immediate family member	mean: 2.1 (minimum 9 months)	Semi-structured interview
Winkler 2019 [90]	Asylum seekers in Berlin	9 refugee shelters	37% Syrian, 15% Afghani, 9% Iraqi, 8% Albanian, 6% Iranian, 5% Moldovan	30.6 (10.0)	M- 75%	NR	NR	128 days (177)	Questionnaire
***Mixed samples of refugees & asylum seekers***:									
Bhui 2006 [26]	Somali refugees in the London Boroughs of Tower Hamlets and Lambeth (UK)	Primary care and community	All Somali	≥18 years	M- 49%	NR	NR	NR	MINI Neuropsychiatric Interview
Leiler 2019 [52]	Adults in asylum accommodations in Jämtland-Härjedalen county	13 asylum accommodations	38% Afghani, 27% Syrian, etc.	71% were <35 years	M- 72%	NR	NR	NR	PHQ-9 (item 9)
Sohn 2019 [77]	Refugees in various stages of the refugee approval process residing in Seoul and Gyeonggi province	Community	51% Sub-Saharan African (Nigerian, Ethiopian, Liberian), 34% Middle Eastern (Yemeni, Egyptian), 8% Asian, etc.	34% <30; 39% 31–40; 21% 41–50; 6% >51	M- 72%	NR	NR	38% <1; 41% 1–5; 21% >5	Korean National Health and Nutrition Examination Survey
Tay 2019 [84]	Rohingya from Myanmar or the offspring of at least one Rohingya parent	49 villages	All Rohingya	28.3 (9.0)	M- 78%	NR	81% tortured, 80% witnessed rape or sexual violence, 69% no food or water, 64% witnessed murder of friends or family, 62% no shelter, 61% witnessed mass killings or atrocities, 56% forced to flee home	47 months (44)	Suicidal ideation was assessed using an item on the depression screen: “how often have you thought about taking your own life in the last two weeks?”
***Internally displaced people***:									
Kim 2007 [49]	Internally displaced persons in Nyala, South Darfur	6 IDP camps	Sudanese	34	Mixed	99% Muslim Sunni, 1% Christian	NR	mean 6.1 months (SE 0.1)	PHQ-9; and yes/no interview questions
Marroquín Rivera 2020 [54]	Adolescents displaced by armed conflict	Community	Colombian	range: 12–17	F- 64%	NR	NR	NR	National Mental Health Survey
Salah 2013 [70]	Internally displaced persons in Khartoum and Gezira IDP areas in Central Sudan	Settlements for IDP	Sudanese	median: 35; range: 18 to 85	M- 44%	NR	Moved due to war/ problem Khartoum: 8.1%; Gezira: 17.5%	Khartoum 18.6 (10.1); Gezira: 18.9 (10.3)	MINI International Neuropsychiatric Interview
Tamayo Martinez 2016 [83]	Adults internally displaced due to conflict	Community	Colombian	mean: 41.2 (95% CI: 39.7, 42.7)	F- 51%	NR	61% exposed to traumatic events themselves or a close person	NR	National Mental Health Survey

*Presented as Mean (SD) unless otherwise noted.

^†^Data were extracted and pooled for the Somali and Kurdish groups as these were reported to be majority refugees in the publication.

^‡^Westman 2006 study’s main countries of birth were, by region, Eastern European: Czechoslovakia, Estonia, Hungary, Romania, Soviet Union; Middle Eastern: Lebanon, Iran, Iraq, Syria, Turkey; Other non-European: Chile, Ethiopia, India, Somalia, Thailand. These regions were extracted since they were reported as mainly refugee countries in the publication.

Abbreviations: BDI = Beck Depression Inventory; F = female; M = male; NR = not reported; PHQ = Patient Health Questionnaire; pub = publication; y = years.

### Suicide

Among the studies using general samples, 11 reported on suicide rates [20, 33, 37, 42, 49, 57, 62, 72, 73, 79, 89]. Suicide results, including a visual bar chart display and some characteristics are presented in Table 2. Six of these studies were in refugees granted asylum, one was in refugees living in a refugee camp, three were in asylum seekers, and one was in internally displaced people. Nine studies were conducted in high income level host countries (HIC; as per 2021 World Bank classification), while the study in a refugee camp was in Thailand (an upper middle-income country, UMIC) and the one study on internally displaced people was in Sudan (a low income country, LIC). The sample size of displaced people in these studies ranged from 4164 to 196,941.

**Table 2:** Suicide rates per 100,000 person-years in general samples, grouped by population type (and then by data source and by host country).

Author, Pub. Year	Population Type	Host Country	Data Source	Study Dates	Suicides, n out of N	Suicide Rate, per 100,000 person-years*
		(Income level)				
Saunders 2017 [72]	Refugees granted asylum	Canada	Vital events registration	1996–2012	n = 27	4.2
		(HIC)			(NR)	
Saunders 2019 [73]	Refugees granted asylum	Canada	Vital events registration	2003–2014	23 out of 58,082	6.1^†^
		(HIC)				
Norredam 2013 [62]	Refugees granted asylum	Denmark	Vital events registration	1994–2007	29 out of 29,139	8^‡^
		(HIC)				
Amin 2021 [20]	Refugees granted asylum	Sweden	Vital events registration	2005–2013^§^	226 out of 196,941	13.2
		(HIC)				
Hollander 2020 [42]	Refugees granted asylum	Sweden	Vital events registration	1991–2015	51 out of 38,123	10^‡^
		(HIC)				
Westman 2006a^¶^ [89]	Refugees granted asylum	Sweden	Vital events registration	1994–1999	35 out of 21,534	25.5^#^
		(HIC)				
Westman 2006b^¶^ [89]	Refugees granted asylum	Sweden	Vital events registration	1994–1999	59 out of 54,643	20.2^#^
		(HIC)				
Westman 2006c^¶^ [89]	Refugees granted asylum	Sweden	Vital events registration	1994–1999	43 out of 34,484	21.4^#^
		(HIC)				
Westman 2006d^¶^ [89]	Refugees granted asylum	Sweden	Vital events registration	1994–1999	27 out of 21,801	20.2^#^
		(HIC)				
Westman 2006e^¶^ [89]	Refugees granted asylum	Sweden	Vital events registration	1994–1999	10 out of 34,940	4.1^#^
		(HIC)				
Westman 2006f^¶^ [89]	Refugees granted asylum	Sweden	Vital events registration	1994–1999	21 out of 35,541	9.4^#^
		(HIC)				
Fellmeth 2016 [33]	Refugees in camps	Thailand	Retrospective cohort	unclear**	4 out of 24,323	16
		(UMIC)				
Goosen 2011a [37]	Asylum seekers	Netherlands	Vital events registration	2002–2007	n = 32 (NR)	25.6
	(Males)	(HIC)				
Goosen 2011b [37]	Asylum seekers	Netherlands	Vital events registration	2002–2007	n = 3 (NR)	4.0
	(Females)	(HIC)				
Staehr 2006 [79]	Asylum seekers	Denmark	Specific monitoring	2003	1 out of 4164	24.0^††^
		(HIC)				
Mittendorfer-Rutz 2020 [57]	Asylum seekers	Sweden	Cross-sectional	2017	12 out of 23,425	51.2
		(HIC)				
Kim 2007 [49]	Internally displaced	Sudan	Cross-sectional	2005	21 out of 7194^‡‡^	290
		(LIC)				

*Crude suicide rate, as the number per 100,000 persons per year, was reported unless otherwise marked.

^†^Saunders 2019 study: 95% CI 3.9, 9.2.

^‡^Rate estimated from estimated follow-up time (i.e., 12 years median in Norredam 2013, and 13 years in Hollander 2020 studies).

^§^The 9-year cohort was extracted for the Amin 2021 study.

^¶^Westman 2006 study’s adjusted results were disaggregated, for gender and birth region, as follows: (a) Eastern European males, (b) Middle Eastern males, (c) Other non-European males, (d) Eastern European females, (e) Middle Eastern females, (f) Other non-European females.

^#^Age-adjusted rate.

**Unclear whether it was 1998–2005 or 1998–2015.

^††^Staehr 2006 study data were extracted for the most recent year reported (i.e., 2003).

^‡‡^Denominator estimated using average reported study household size.

Abbreviations: HIC = high income country; LIC = low income country; NR = not reported; pub = publication; UMIC = upper middle income country.

All six studies in refugees granted asylum and one of the studies on asylum seekers used vital events registration as the data source. The other studies on asylum seekers used specific monitoring data as the data source or a cross-sectional study design. The study in a refugee camp was a retrospective cohort, and the one on internally displaced people used a cross-sectional study design.

Suicide rates ranged considerably from 4.0 to 290 per 100,000 person-years across the 11 studies in general samples (Table 2). In refugees granted asylum, suicide rates ranged from 4.1 to 25.5 per 100,000 person-years, while in asylum seekers suicide rates ranged from 4.0 to 51.2 per 100,000 person-years. The study in a refugee camp found a suicide rate of 16 per 100,000 person-years, while the study on internally displaced people (in Sudan, a LIC) had the highest suicide rate of 290 per 100,000 person-years.

**Fig 2:**
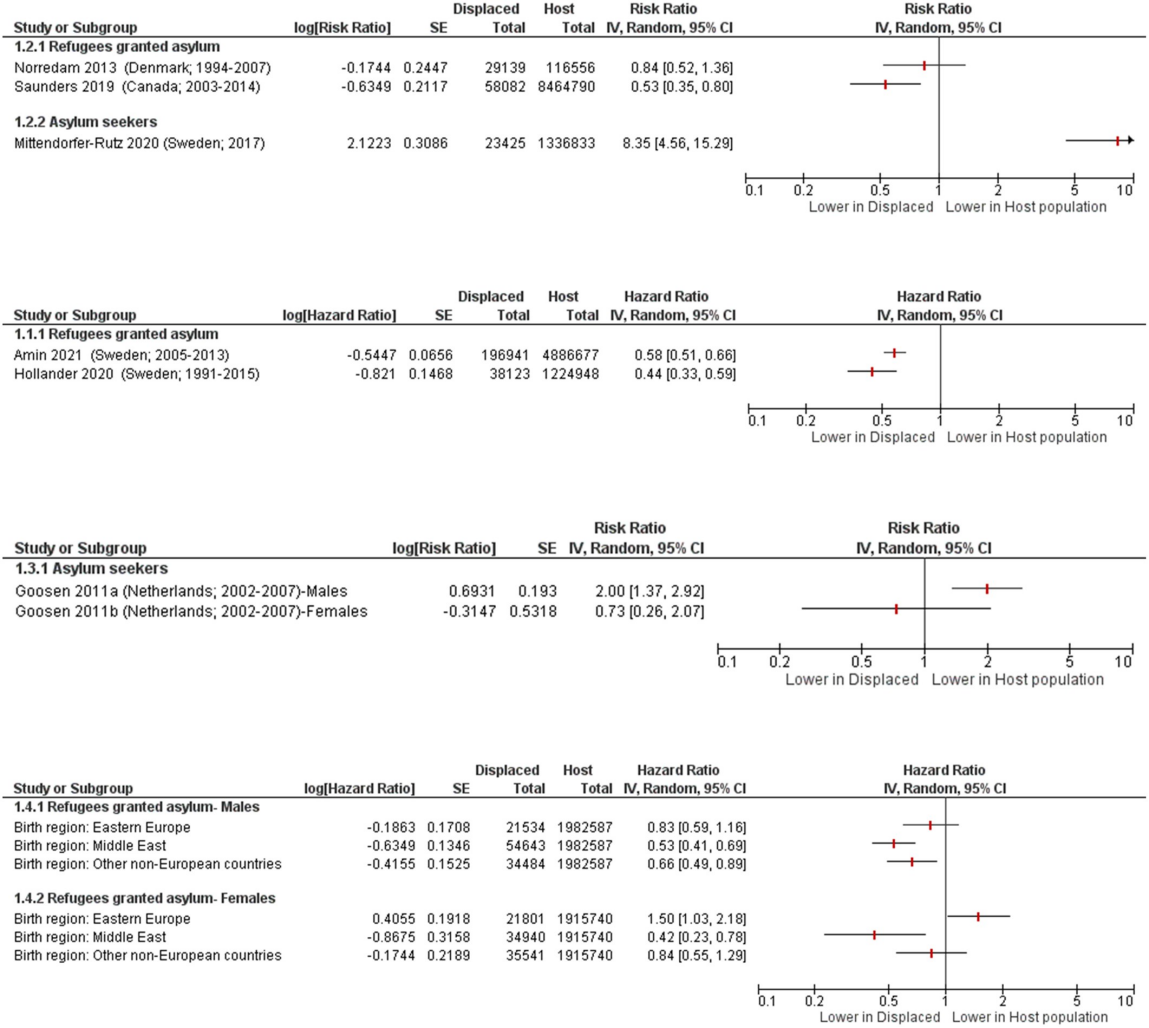
Suicide in displaced people compared to host country population. Norredam 2013 [62] adjusted for age and income; Amin 2021 [20] adjusted for socio-demographic, labour market marginalisation and morbidity factors (i.e. sex, age, educational level, family situation and type of residential area, days with full-time unemployment, net days with sickness absence, granted disability pension, history of suicide attempt; history of inpatient or specialised outpatient healthcare); Hollander 2020 [42] adjusted for attained age, gender, and disposable income; Goosen 2011 [37] standardised for age; Westman 2006 [89] adjusted for age, marital status, socioeconomic status, and hospitalization for psychiatric disorders or substance abuse; Saunders 2019 [73] and Mittendorfer-Rutz 2020 [57] were unadjusted.

Only eight studies with general samples also compared suicide rates in displaced people to the host population, including five studies in refugees granted asylum and three conducted in asylum seekers. Seven studies [20, 37, 42, 57, 62, 72, 89] reported data in formats that could be presented visually in forest plots, which were grouped by outcome measure and then sub-grouped by population type (Fig 2).

Among the studies in refugees granted asylum, there was evidence of a lower risk of suicide compared with the host population in four studies [20, 42, 62, 72]. The final study on refugees granted asylum [89] reported lower, higher, and inconclusive risk compared with the host population depending on gender of the refugees and their birth region. In contrast, among the studies in asylum seekers there was evidence of a higher suicide risk compared with the host population in two studies [50, 57], including one study that reported age-standardised mortality ratios (SMR) of 2.8 [95% CI 1.5, 4.1] in males, and 1.5 [95% CI 0.0, 3.6] in females [50]. The third asylum seekers study was inconclusive about whether the risk was higher or lower, as it varied based on gender [37].

An additional study [31] on asylum seekers in detention in the UK estimated 211 suicides per 100,000, however, a rate estimate could not be calculated as total person-year was not reported.

Only two studies using specific samples were identified that reported on suicide rates; one study described forensic autopsies of deceased refugees [36], and the other study only reported on the proportion of suicides amongst causes of death in refugees [41] (S3 Table).

### Suicide attempt

Among the studies using general samples, 15 [17, 18, 20, 30, 39, 47–49, 54, 55, 77, 79, 83, 85, 88] reported on the prevalence of suicide attempts (Table 3). The results are ordered by timeframe for the prevalence estimates and then sub-grouped by population type. Six of these studies were in refugees granted asylum, three were in refugees living in refugee camps, one was in mixed refugees & asylum seekers, two were in asylum seekers, and three were in internally displaced people. Ten studies were from high income countries, and the other five were conducted in India (a lower middle-income country, LMIC), Jordan (UMIC), Sudan (LIC), and two in Colombia (UMIC). The sample sizes of displaced people ranged from 53 to 196,941.

**Table 3:** Suicide attempt prevalence percentage in general samples, ordered by timeframe (and then by population type).

Author, Pub. Year	Population Type	Host Country	Data Source	Study Dates	Suicide Attempts, n out of N	Time-frame*	Prevalence^†^, %
		(Income level)					(95% CI)^‡^
Amin 2021 [20]	Refugees granted asylum	Sweden	Prospective cohort	2005–2013^§^	1607 out of 196,941	9 years	0.82^¶^
		(HIC)					(0.78 to 0.86)
Vijayakumar 2017 [88]	Refugees in camps	India	Cross-sectional	NR	11 out of 1755	15 months	0.63^#^
		(LMIC)					(0.31 to 1.1)
Tousignant 1999 [85]	Refugees granted asylum	Canada	Cross-sectional	NR	7 out of 203	1 year	3.5**
		(HIC)					(1.4 to 7.0)
Choi 2020 [30]***	Refugees granted asylum	South Korea	Cross-sectional	2011–2018	13 out of 86	1 year	15.1^††^
							(8.3 to 24.5)
Staehr 2006 [79]	Asylum seekers	Denmark	Specific monitoring	2003	43 out of 4164	1 year	1.0^‡‡^
		(HIC)					(0.75 to 1.4)
Sohn 2019 [77]***	Mixed populations	South Korea	Cross-sectional	NR	1 out of 129	1 year	0.78^§§^
							(0.02 to 4.2)
Kim 2007 [49]	Internally displaced	Sudan	Cross-sectional	2005	28 out of 1260	1 year	2.2
		(LIC)					(1.5 to 3.2)
Keller 2003 [48]	Asylum seekers	USA	Cross-sectional	2001–2002	2 out of 70	5 months^¶¶^	2.9
		(HIC)					(0.35 to 9.9)
Hermans 2017 [39]	Refugees in camps	Greece	Prospective cohort	2016	4 out of 2291	6 weeks	0.18^##^
		(HIC)					(0.05 to 0.45)
Jankovic 2013 [47]***	Refugees granted asylum	UK, Italy, Germany	Cross-sectional	2005–2006	6 out of 854	1 month	0.70
							(0.26 to 1.5)
Meyerhoff 2020 [55]	Refugees granted asylum	USA	Cross-sectional	NR	4 out of 53	1 week	7.6
		(HIC)					(2.1 to 18.2)
Alley 1982 [18]	Refugees granted asylum	USA	Cross-sectional	1980	6 out of 4192	NR	0.14
		(HIC)					(0.05 to 0.31)
Al-Modallal 2012 [17]	Refugees in camps	Jordan	Cross-sectional	NR	21 out of 287	NR	7.3
		(UMIC)					(4.6 to 11.0)
Tamayo Martinez 2016 [83]	Internally displaced	Colombia	National statistics	2015	N = 943 surveyed^†††^	NR	5.5
		(UMIC)					(3.1 to 9.6)
Marroquín Rivera 2020 [54]	Internally displaced	Colombia	Cross-sectional	2015	9 out of 99	NR	9.1
		(UMIC)					(4.2 to 16.6)

*Timeframe refers to either how far back participants were asked about the occurrence of a suicide attempt (for example, in the past year), or the follow-up time.

^†^Prevalence rate per 100,000 person-years (i.e., the number per 100,000 persons per year) is also presented when it was either reported or it could be estimated from study follow-up time.

^‡^Confidence intervals (of prevalence percentages) were calculated using Stata 12.

^§^The 9-year cohort was extracted for the Amin 2021 study.

^¶^Amin 2021 study’s reported rate is 94.2 per 100,000 person-years.

^#^Vijayakumar 2017 study’s estimated rate is 500 per 100,000 person-years.

**Tousignant 1999 study’s estimated rate is 3450 per 100,000 person-years.

^††^Choi 2020 study’s estimated rate is 15,100 per 100,000 person-years.

^‡‡^Staehr 2006 study’s reported rate is 1032 per 100,000 person-years (for the most recent year reported, i.e., 2003).

^§§^Sohn 2019 study’s estimated rate is 775 per 100,000 person-years.

^¶¶^Median 5 months (range 1–54).

^##^Hermans 2017 study’s reported rate is 1400 per 100,000 person-years (95% CI, 30 to 2800).

***High income countries (HIC).

^†††^Absolute number of suicide attempts was not reported.

Abbreviations: A.S. = asylum seekers; HIC = high income country; LIC = low income country; LMIC = lower middle income country; NR = not reported; pub = publication; Ref. = refugees; UMIC = upper middle income country.

Two of the 15 studies had a prospective cohort design, one used specific monitoring data as the data source, one used national statistics, while 11 studies had a cross-sectional design. The prevalence timeframe of the studies (i.e. either how far back participants were asked about the occurrence of a suicide attempt or the follow-up time) ranged from 1 week to 9 years.

The prevalence of suicide attempts ranged from 0.14% to 15.1% across the studies in general samples (Table 3). In refugees granted asylum, the prevalence of suicide attempt ranged from 0.14% to 15.1%, and in the refugees living in camps it ranged from 0.18% to 7.3%. In the studies on asylum seekers the prevalence was 1.0% and 2.9%, and in internally displaced people it ranged from 2.2% to 9.1%. The study in mixed refugees & asylum seekers found a suicide attempt prevalence of 0.78%. The one study in a LIC (Sudan) did not find a higher prevalence than average.

**Fig 3:**
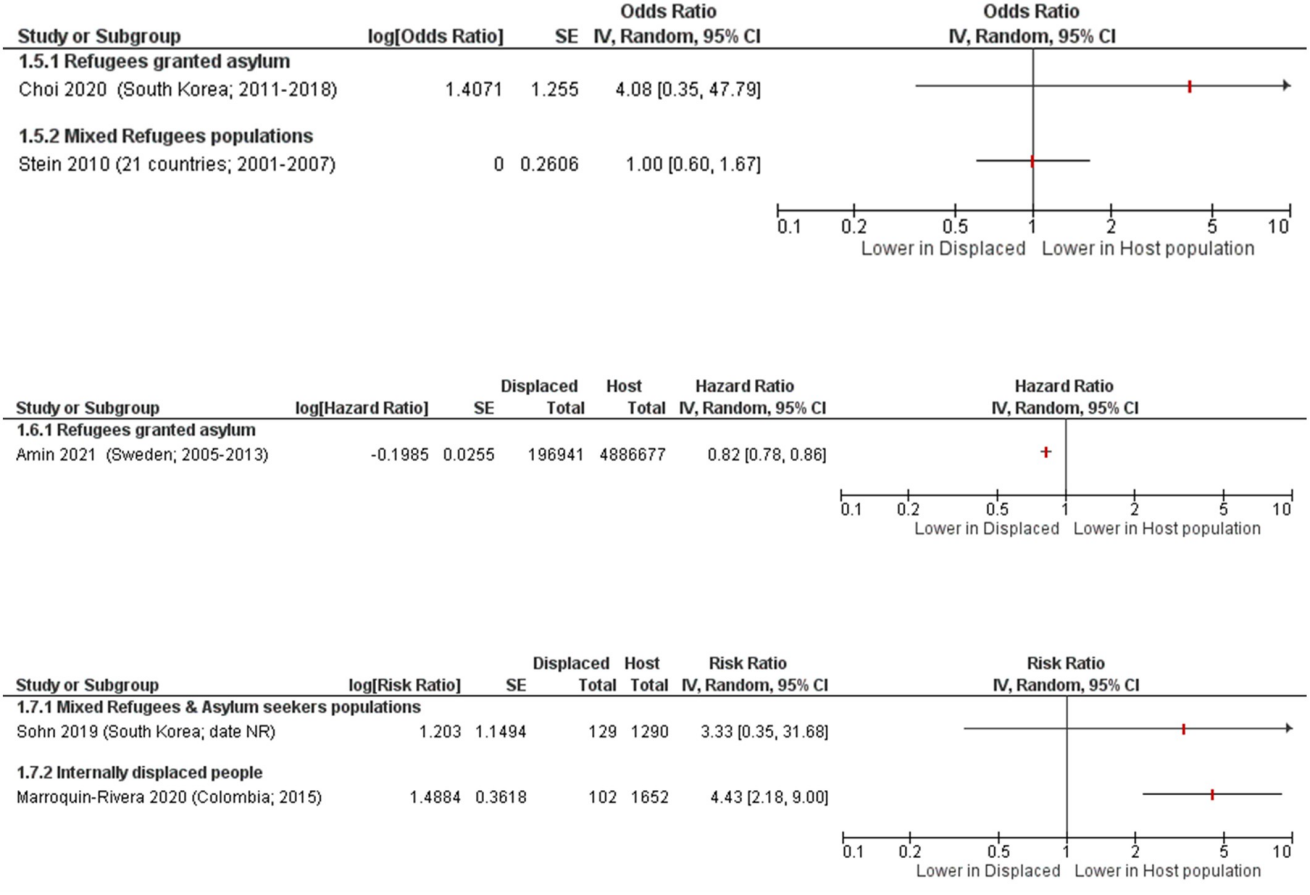
Suicide attempts in displaced people compared to host population forest plots, grouped by outcome measure and then sub-grouped by population type. Choi 2020 [30] adjusted for interview year; Stein 2010 [81] adjusted for person-year, country, demographic factors (age, sex, time-varying education, time-varying marriage), interactions between life course and demographic variables, parent psychopathology, and childhood adversities; Amin 2021 [20] adjusted for socio-demographic, labour market marginalisation and morbidity factors (i.e. sex, age, educational level, family situation and type of residential area, days with full-time unemployment, net days with sickness absence, granted disability pension, history of suicide attempt; history of inpatient or specialised outpatient healthcare); Sohn 2019 [77] age- and gender-matched; Marroquin-Rivera 2020 [54] unadjusted.

Six studies in general samples compared the prevalence of suicide attempts in displaced people to the host population. Only five of these studies [20, 30, 54, 77, 81] reported data in formats that could be presented visually in forest plots (Fig 3). Two of these studies were in refugees granted asylum, one was in mixed refugees & asylum seekers, one was in internally displaced people, and one was a pooled report of mixed refugee populations across 21 countries [81].

In the two studies on refugees granted asylum, one reported a lower risk of suicide attempt than in the host population [20] while the other reported a higher risk [30]. The study of mixed refugee types [81] reported an odds ratio of 1.00 [95% CI 0.60, 1.67] indicating little to no difference between the refugees and the host populations. In one study [77] on mixed refugees & asylum seekers, they found a higher risk of suicide attempts. One study [54] on internally displaced people found a 4-fold greater risk of suicide attempt compared to the host population, RR 4.43 [95% CI 2.18, 9.00]. In addition, one study [79] found asylum seekers had a 3.4 times higher rate of suicide attempts compared to the host population (unadjusted).

One study [87] reported a composite outcome of self-harm or suicide. Refugees granted asylum were evaluated over a one-year timeframe and prevalence was reported as 0.10%, the rate was 101.2 per 100,000 person-years, and the comparative result was RR 0.80 [95% CI 0.58, 1.08] (unadjusted) for self-harm or suicide compared to the host population.

An additional three studies reported only a composite outcome of self-harm or suicide attempt. One [72] was conducted in refugees granted asylum, and the study timeframe ranged from 5–14 years. Prevalence was 0.68%, and the rate was 8.09 per 100,000 person-years [95% CI 7.31, 8.93]. The comparative result was found to be RR 0.85 [95% CI 0.77, 0.94] (unadjusted) for this composite outcome. In contrast, one of two studies [37] was conducted in asylum seekers, and the study timeframe was not reported. The rate was 119.2 per 100,000 person-years in males, and 188.2 per 100,000 person-years in females. Compared to the host population for self-harm or suicide attempt, asylum seekers had age-standardised RR 1.42 [95% CI 1.20, 1.66] in males, and RR 1.00 [95% CI 0.84, 1.18] in females. The other study [80] in asylum seekers assessed 10 detained families and found the prevalence of self-harm or suicide attempt was 29.4%.

Nine studies using specific samples of the target populations reported on suicide attempts (S4 Table).

### Suicidal ideation

Among the studies with general samples, 37 [16–18, 21, 25, 30, 32, 35, 44, 45, 47–49, 52, 54, 55, 60, 61, 64, 66, 70, 71, 75, 77–80, 82–84, 86, 88, 90] reported on the prevalence of suicidal ideation (Table 4). The findings were ordered by prevalence timeframe and sub-grouped by population type. 11 studies were in refugees granted asylum, 11 were in refugees living in refugee camps, eight were in asylum seekers, three were in mixed refugees & asylum seekers, and four were in internally displaced people. 22 studies were from high income countries, and 15 were conducted in: India (LMIC), Lebanon (UMIC), Malaysia (UMIC), Nigeria (LMIC), Occupied Palestinian Territory (LMIC), Pakistan (LMIC), Syria (LIC), Thailand (UMIC), Uganda (LIC), and two each in Colombia (UMIC), Jordan (UMIC), and Sudan (LIC).

**Table 4:** Suicidal ideation prevalence percentage in general samples, ordered by timeframe (and then by population type).

**Author, Pub. Year**	**Population Type**	**Host Country** *	**Data Source**	**Study Dates**	**Suicidal Ideation, n out of N**	**Time-frame** ^†^	**Prevalence** ^‡^ **, %**
		**(Income level)**					**(95% CI)** ^§^
Park 2018 [64]	Refugees granted asylum	South Korea	Specific monitoring	2017–2018	29 out of 174	1 year	16.7^¶^
		(HIC)					(11.5 to 23.1)
Choi 2020 [30]	Refugees granted asylum	South Korea	Cross-sectional	2011–2018	24 out of 86	1 year	27.9^#^
		(HIC)					(18.8 to 38.6)
Noh 2017 [61]	Refugees granted asylum	South Korea	Cross-sectional	2008–2014	153 out of 656	1 year	23.3**
		(HIC)					(20.1 to 26.8)
Um 2020 [86]	Refugees granted asylum	South Korea	Cross-sectional	2014	127 out of 405	1 year	31.4^††^
		(HIC)					(26.9 to 36.1)
Ao 2016 [21]	Refugees granted asylum	USA	Cross-sectional	2008–2011	9 out of 423	1 year	2.1^‡‡^
		(HIC)					(1.0 to 4.0)
Itani 2017a [45]^¶¶¶^	Refugees in camps	Occupied Palestinian T.	National statistics	2010	2257 out of 8526	1 year	26.5^§§^
							(25.5 to 27.4)
Itani 2017b [45]	Refugees in camps	Jordan	Cross-sectional	2010	404 out of 1495	1 year	27.0^§§^
		(UMIC)					(24.8 to 29.4)
Itani 2017c [45]	Refugees in camps	Lebanon	Cross-sectional	2010	431 out of 2168	1 year	19.9^§§^
		(UMIC)					(18.2 to 21.6)
Itani 2017d [45]	Refugees in camps	Syria	Cross-sectional	2010	567 out of 2114	1 year	26.8^§§^
		(LIC)					(24.9 to 28.8)
Staehr 2006 [79]	Asylum seekers	Denmark	Specific monitoring	2003	7 out of 4164	1 year	0.17^¶¶^
		(HIC)					(0.07 to 0.35)
Sohn 2019 [77]	Mixed Ref. & A.S. populations	South Korea	Cross-sectional	NR	4 out of 129	1 year	3.1^##^
		(HIC)					(0.85 to 7.8)
Kim 2007 [49]	Internally displaced	Sudan	Cross-sectional	2005	66 out of 1257	1 year	5.3***
		(LIC)					(4.1 to 6.6)
Keller 2003 [48]	Asylum seekers	USA	Cross-sectional	2001–2002	18 out of 70	5 months^†††^	25.7
		(HIC)					(16.0 to 37.6)
Jankovic 2013 [47]	Refugees granted asylum	UK, Italy, Germany (HIC)	Cross-sectional	2005–2006	81 out of 854	1 month	9.5
							(7.6 to 11.7)
Akinyemi 2012 [16]	Refugees in camps	Nigeria	Cross-sectional	2010	49 out of 444	1 month	11.0
		(LMIC)					(8.3 to 14.3)
Rahman 2003 [66]	Refugees in camps	Pakistan	Cross-sectional	2002	96 out of 297	1 month	32.3
		(LMIC)					(27.0 to 38.0)
Falb 2013 [32]	Refugees in camps	Thailand	Cross-sectional	2008	63 out of 848	1 month	7.4
		(UMIC)					(5.8 to 9.4)
Nickerson 2019a [60]	Refugees granted asylum	Australia	Cross-sectional	2015–2018	12 out of 826	2 weeks	1.5
		(HIC)					(0.75 to 2.5)
Vijayakumar 2017 [88]	Refugees in camps	India	Cross-sectional	NR	16 out of 1303	2 weeks	1.2
		(LMIC)					(0.70 to 2.0)
Nickerson 2019b [60]	Asylum seekers	Australia	Cross-sectional	2015–2018	21 out of 259	2 weeks	8.1
		(HIC)					(5.1 to 12.1)
Leiler 2019 [52]	Mixed Ref. & A.S. populations	Sweden	Cross-sectional	2016–2017	173 out of 510	2 weeks	33.9
		(HIC)					(29.8 to 38.2)
Salama 2020 [71]	Refugees granted asylum	Finland	Cross-sectional	2010–2012	80 out of 850	1 week	9.4
		(HIC)					(7.5 to 11.6)
Meyerhoff 2020 [55]	Refugees granted asylum	USA	Cross-sectional	NR	4 out of 60	1 week	6.7
		(HIC)					(1.9 to 16.2)
Fuhrer 2016 [35]	Asylum seekers	Germany	Cross-sectional	2015	12 out of 214	1 week	5.6
		(HIC)					(2.9 to 9.6)
Huemer 2011 [44]	Asylum seekers	Austria	Cross-sectional	NR	4 out of 41	current state	9.8
		(HIC)					(2.7 to 23.1)
Steel 2004 [80]	Asylum seekers	Australia	Cross-sectional	2002–2003	24 out of 34	current state	70.6
		(HIC)					(52.5 to 84.9)
Sultan 2001 [82]	Asylum seekers	Australia	Cross-sectional	2001	23 out of 33	current state	69.7
		(HIC)					(51.3 to 84.4)
Bhui 2003 [25]	Refugees granted asylum	UK	Cross-sectional	NR	62 out of 177	NR	35.0
		(HIC)					(28.0 to 42.5)
Alley 1982 [18]	Refugees granted asylum	USA	Cross-sectional	1980	10 out of 4192	NR	0.24
		(HIC)					(0.12 to 0.44)
Al-Modallal 2012 [17]	Refugees in camps	Jordan	Cross-sectional	NR	39 out of 287	NR	13.6
		(UMIC)					(9.9 to 18.1)
Slodnjak 2002 [75]	Refugees in camps	Slovenia	Cross-sectional	1994	64 out of 265	NR	24.1
		(HIC)					(19.1 to 29.8)
**Author, Pub. Year**	**Population Type**	**Host Country** *	**Data Source**	**Study Dates**	**Suicidal Ideation, n/N**	**Time-frame** *	**Prevalence** ^†^ **, %**
		**(Income level)**					**(95% CI)** ^‡^
Ssenyonga 2012 [78]	Refugees in camps	Uganda	Cross-sectional	NR	89 out of 426	NR	20.9
		(LIC)					(17.1 to 25.1)
Winkler 2018 [90]	Asylum seekers	Germany	Cross-sectional	2015–2016	87 out of 496	NR	17.5
		(HIC)					(14.3 to 21.2)
Tay 2019 [84]	Mixed Ref. & A.S. populations	Malaysia	Cross-sectional	NR	228 out of 959	NR	23.8
		(UMIC)					(21.1 to 26.6)
Tamayo Martinez 2016 [83]	Internally displaced	Colombia	National statistics	2015	N = 943 surveyed^§§§^	NR	12.5
		(UMIC)					(9.0 to 17.1)
Marroquín Rivera 2020 [54]	Internally displaced	Colombia	Cross-sectional	2015	20 out of 101	NR	19.8
		(UMIC)					(12.5 to 28.9)
Salah 2013 [70]	Internally displaced	Sudan	Cross-sectional	2008	9 out of 1876	NR	0.48
		(LIC)					(0.22 to 0.91)

*Refers to Country or Territory as applicable.

^†^Timeframe refers to either how far back participants were asked about the occurrence of suicidal ideation (for example, in the past year), or the follow-up time.

^‡^Prevalence rate per 100,000 person-years (i.e., the number per 100,000 persons per year) is also presented when it was either reported or it could be estimated from study follow-up time.

^§^Confidence intervals (of prevalence percentages) were calculated using Stata 12.

^¶^Park 2018 study’s estimated rate is 16,700 per 100,000 person-years.

^#^Choi 2020 study’s estimated rate is 27,900 per 100,000 person-years.

**Noh 2017 study’s estimated rate is 23,300 per 100,000 person-years.

^††^Um 2020 study’s estimated rate is 31,400 per 100,000 person-years.

^‡‡^Ao 2016 study’s estimated rate is 2100 per 100,000 person-years.

^§§^Itani 2017a, 2017b, 2017c, and 2017d studies’ estimated rates are, per 100,000 person-years: 26,500, 27,000, 19,900, and 26,800 (respectively).

^¶¶^Staehr 2006 study’s reported rate is 168 per 100,000 person-years (for the most recent year reported, i.e., 2003).

^##^Sohn 2019 study’s estimated rate is 3100 per 100,000 person-years.

***Kim 2007 study’s estimated rate is 5300 per 100,000 person-years.

^†††^Median was 5, range 1–54 months.

^§§§^Absolute number having suicidal ideation was not reported.

^¶¶¶^Lower middle income (as per World Bank classification, 2021).

Abbreviations: A.S. = asylum seekers; HIC = high income country; LIC = low income country; LMIC = lower middle income country; NR = not reported; pub = publication; Ref. = refugees; T. = Territory; UMIC = upper middle income country.

Two of the 37 studies used specific monitoring data as the data source, two used national statistics, and 33 had a cross-sectional design. The timeframe of the evaluation ranged from the ‘current state’ to 1 year. Sample sizes of displaced people ranged from 33 to 8526.

Prevalence of suicidal ideation ranged from 0.17% to 70.6% across the studies in general samples (Table 4). In refugees granted asylum, the prevalence of suicidal ideation ranged from 0.24% to 35.0%, and in refugees living in camps it ranged from 1.2% to 32.3%. In asylum seekers the prevalence ranged from 0.17% to 70.6%, and in internally displaced people from 0.48% to 19.8%. The mixed refugees & asylum seekers studies reported prevalences of 3.1%, 23.8% and 33.9%. There was not a pattern of generally higher prevalence in low income countries across the 4 LIC studies.

**Fig 4:**
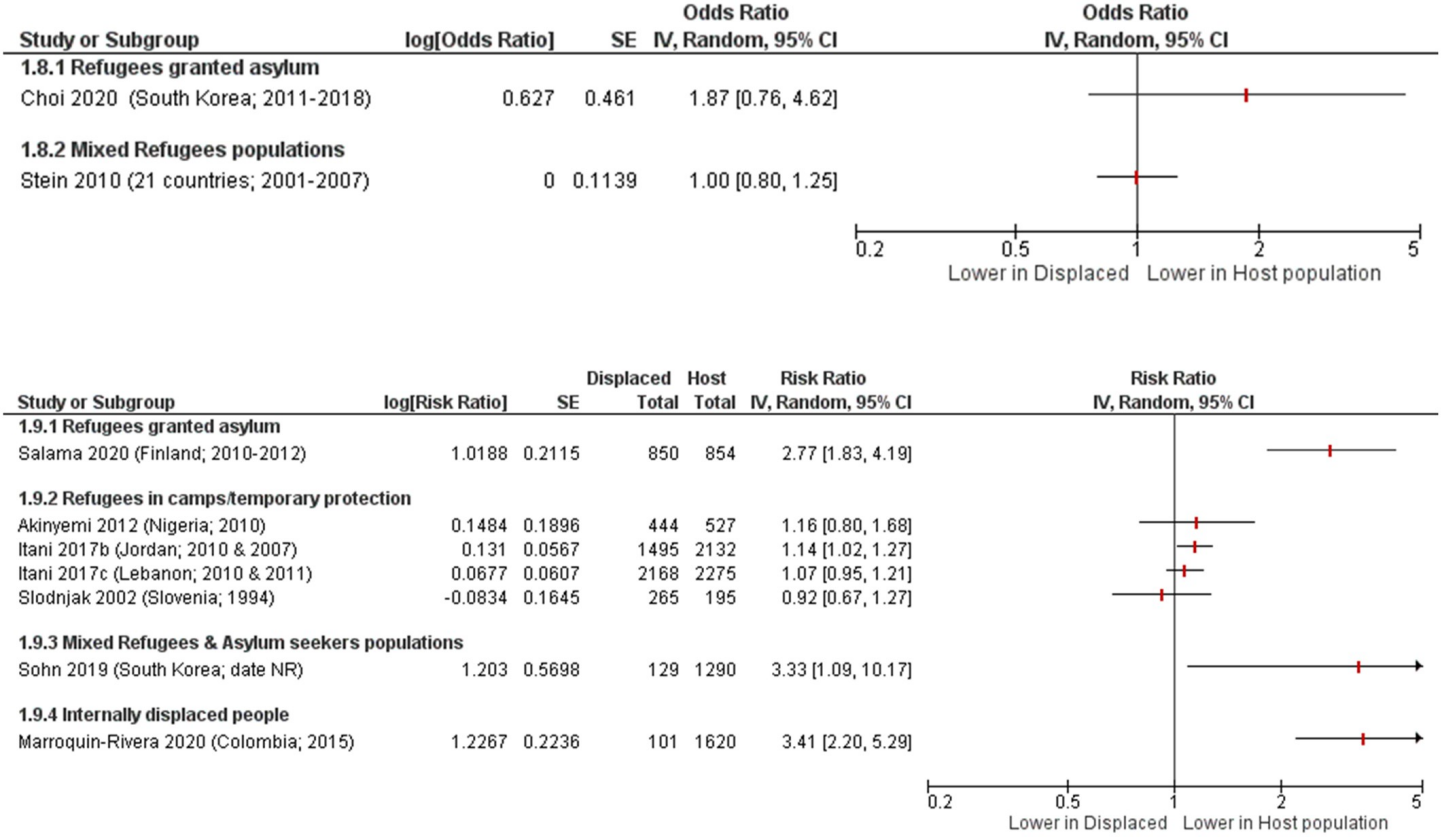
Suicidal ideation in displaced people compared to host population forest plots, grouped by outcome measure and then by population type. Choi 2020 [30] adjusted for interview year; Stein 2010 [81] adjusted for person-year, country, demographic factors (age, sex, time-varying education, time-varying marriage), interactions between life course and demographic variables, parent psychopathology, and childhood adversities; Sohn 2019 [77] age- and gender-matched; Akinyemi 2012 [16], Itani 2017 [45], Slodnjak 2002 [75], Marroquin-Rivera 2020 [54], and Salama 2020 [71] were unadjusted.

Only nine studies [16, 30, 45, 54, 71, 75, 77, 81] in general samples compared suicidal ideation in displaced people to the host population as displayed in Fig 4. Two of these studies were in refugees granted asylum, four were in refugees living in refugee camps with only temporary protection, one was in mixed refugees & asylum seekers, one was in internally displaced people, and one was a pooled report of mixed refugees across 21 countries [81].

Among refugees living in refugee camps, there was evidence of a higher risk of suicidal ideation compared with the host population from three studies [16, 45], while one study reported a lower risk [75]. There was evidence of a higher risk of suicidal ideation among refugees granted asylum in two studies [30, 71]. Individual studies reported higher risk of suicidal ideation in mixed refugee and asylum seeker populations [77], and internally displaced people [54]. The report [81] of mixed refugees across 21 countries reported little to no difference in risk of suicidal ideation compared with the host population (OR 1.00 [95% CI 0.80, 1.25]).

An additional two studies in general samples reported a composite outcome of suicidal ideation or suicide attempt. One study [26] evaluated mixed refugees & asylum seekers, and the study timeframe was 1 month. Prevalence was 9.1% [95% CI 4.9, 15] for suicidal ideation or suicide attempt. The other [29] was conducted in asylum seekers, and the prevalence was 55.6% for this composite outcome. This study’s timeframe was not reported.

Three studies only reported continuous outcome data on suicidal ideation. One [76] was conducted in refugees granted asylum but the study’s timeframe was not reported. Mean score using the Suicidal Ideation Scale was 20.34 (SD 7.39). A study [91] in refugees with only temporary protection reported mean scores using the Personality Assessment Index’s Suicide Ideation subscale were 49.5 (SD 10.9) in males and 53.8 (SD 17.0) in females (timeframe was not reported). The other [38] assessed asylum seekers, and the timeframe was 2 weeks. Mean score using the PHQ-9 symptom severity Item 9 for suicidal ideation was 0.98 (SD 1.11).

Sixteen studies using specific samples reported on suicidal ideation (S5 Table).

## Discussion

This systematic review identified 77 studies that reported on suicide, suicide attempts, or suicidal ideation in displaced people. Estimates of suicide rates from the studies which are more representative of refugee and asylum seeker populations (i.e. general samples) ranged widely and appeared to differ based on the source of the data and the population subgroup being assessed. Estimates of the prevalence of suicide attempts or suicidal ideation also ranged widely and were inconsistently reported across studies, making comparisons across groups challenging.

While numerous studies have been published on this topic, the evidence base is still sparse for the differing contexts of refugees in refugee camps, asylum seekers, and internally displaced people. Additionally, fewer than half of the studies reported on suicides or suicide attempts and almost one third of the studies were not representative samples of the target population. This systematic review has provided an overview of the populations, contexts, and study designs that have been used for research on suicide and suicidal behaviours in these populations, but further research is warranted to fill the gaps in knowledge around these specific populations.

The heterogeneity in study populations, designs, analytical approaches, and results mean that it is not possible to draw firm conclusions on the comparative rate of suicide in refugees and host populations. The results from different studies are inconclusive, with several studies suggesting that rates of suicide may be lower in refugees granted asylum than in the host populations [20, 42, 72, 89]. These results should be interpreted with caution because the variability of the available data may be due to effect estimates being mediated by a range of mechanisms. These factors might include the local context (such as rates or prevalence of suicidal behaviour in the host population, as well as possible confounders such as poverty and deprivation), as well as possible mechanisms of action, such as the extent of physical injury, loss of housing, financial distress, and the level of social support that is provided. The length of stay in a host country has also been suggested as a potential mediator, with refugees having a lower risk once they have lived in a host country for longer than 10 years [42].

The strengths of this systematic review included developing a sensitive search strategy and screening all results in duplicate, as well as careful evaluation and reporting of the data according to study design and population. However, there are some limitations to this review, particularly around the sparse international evidence base which prevents us from drawing firm conclusions. The wide variety of study designs and approaches to reporting risks prevented meta-analysis of the results. This variety in study designs also means that we were only able to do a relatively high-level assessment of methodological quality based on the source of data for the studies included in this review. Further data on mediators or risk factors for suicidal behaviour in these populations could add to the evidence base and strengthen the development of prevention or management interventions. In addition, there is potential for overlap in the five population subgroups used in this review. It is often difficult to distinguish which of these subgroups a study population belongs to without access to further population characteristics from the primary studies.

Despite the sparsity of studies, there is evidence of a high risk of suicide and suicidal behaviour among refugees in camps and asylum seekers, which suggests that these groups require additional support and monitoring. Strengthening the evidence base around the risk of suicidal behaviour in these vulnerable groups is needed to further develop understanding of the factors that influence them. Many factors have been suggested to influence the risk of suicide and suicidal ideation in these populations, including a combination of socioeconomic disadvantage [93], exposure to potentially traumatic events [103], increased depression and anxiety [104], or a lack of appropriate and accessible care [39, 105]. Improvements in understanding the suicide risk in these populations can be made by better routine data collection to enable consistent surveillance across countries. Countries hosting people affected by conflict, including refugees and asylum seekers, should ensure that these groups are identifiable in routine data [12, 104]. Agreed methods for analysing and presenting data would support comparisons between exposed populations in different countries and settings and would aid in quantitative synthesis of the risk of suicidal behaviours.

Finally, a better understanding of mediating factors may also help to inform policy responses and potential interventions for refugees or displaced people. A recent review found only a limited number of suicide prevention or response programs were implemented for refugee or displaced populations [4]. The review recommended programs that were multi-tiered, focussing on multiple levels of suicide prevention. A comprehensive approach to suicide prevention should include adequate surveillance, identify risk and protective factors, and ensure evaluation of programs following implementation. Studies on prevalence are well placed to identify risk and protective factors that might explain some of the variability across populations and contexts. For example, studies should consider including measures of local context as well as potential mediators such as experience of physical injury, loss of housing, financial hardship, levels of social support, legal context, and access to mental health services [106, 107].

Given the high risk of suicide in refugees in camps and asylum seekers, host countries should increase efforts to prevent suicide in asylum seekers, with actions that may span from speeding up asylum processes and improving social conditions of asylum seekers to offering better mental health prevention and management interventions. Given that prevalence of mental disorders is high in displaced people and that that these conditions appear associated with suicide attempts, there is a need to invest in prevention and care of mental disorders in this vulnerable group.

## Supporting information

S1 Table PubMed search strategy to identify studies on suicide in displaces people. (PDF)

S2 Table Characteristics of included studies using specific samples, grouped by population type. (PDF)

S3 Table Suicide rates per 100,000 person-years using specific samples. (PDF)

S4 Table Suicide attempt prevalence percentage using specific samples, ordered by population type (and then by author). (PDF)

S5 Table Suicidal ideation prevalence percentage using specific samples, ordered by population type (and then by author). (PDF)

S1 Checklist PRISMA 2020 checklist. (PDF)
