# Supplementary material for: Suicide rates and suicidal behaviour in displaced people: A systematic review
Source: PLoS One. 2022 Mar 10;17(3):e0263797. doi: 10.1371/journal.pone.0263797 (PMC8912254; doi:10.1371/journal.pone.0263797)
Supplement: S1 Table — (PDF) [file pone.0263797.s001.pdf]

S1 Table. PubMed search strategy to identify studies on suicide in displaced people.

Search	Query
#1	Search suicide or suicidal Field: Title/Abstract
#2	Search self poisoning or self harm* or self-injury or parasuicid* or suicidality Field: Title/Abstract
#3	Search (#1) OR #2
#4	Search "Suicide, Attempted"[Mesh] OR "Suicide"[Mesh]
#5	Search "Suicidal Ideation"[Mesh]
#6	Search "Self-Injurious Behavior"[Mesh]
#7	Search (("Mental Health/statistics and numerical data"[Mesh]))
#8	Search ((((#7) OR #6) OR #5) OR #4) OR #3
#9	Search refugee* Field: Title/Abstract
#10	Search "Refugees"[Mesh] OR "Refugee Camps"[Mesh]
#11	Search migrant* or "displaced person*" or "displaced people" Field: Title/Abstract
#12	Search camp OR camps OR settlement OR shelter OR accommodation Field: Title/Abstract
#13	Search "Human Migration"[Mesh]
#14	Search displacement Field: Title/Abstract
#15	Search asylum seeker* Field: Title/Abstract
#16	Search "Natural Disasters"[Mesh]
#17	Search ("Emigrants and Immigrants"[Mesh] OR "Emigration and Immigration"[Mesh])
#18	Search (((((((#17) OR #16) OR #15) OR #14) OR #13) OR #12) OR #10) OR #9)
#19	Search (#18) AND #8
