# Supplementary material for: Suicide rates and suicidal behaviour in displaced people: A systematic review
Source: PLoS One. 2022 Mar 10;17(3):e0263797. doi: 10.1371/journal.pone.0263797 (PMC8912254; doi:10.1371/journal.pone.0263797)
Supplement: S2 Table — (PDF) [file pone.0263797.s002.pdf]

S2 Table. Characteristics of included studies using specific samples, grouped by population type

Author, Pub. Year	Selection Criteria/ Study Denominator	Setting	Ethnicity of Displaced People	Age (y)*	Gender	Religion	Trauma Exposure	Duration of Resettlement	Data Collection Source or Measurement
Refugees granted permanent asylum status:									
Ferrada-Noli 1996a (34)	Patients with diagnosed PTSD	Psychiatric clinics	27% Iranian, 19% Latin American, 14% Lebanese, 8% Iraqi, 6% Bangladeshi	Mean: 35	M- 91%	55% Muslim, 30% Christian	All traumatised	NR	Clinical assessment (checklist)
Gleich 2018 (36)	Forensic autopsies of deceased refugees ordered by the prosecuting attorney's office in Munich	Institute of Forensic Medicine	25% Afghani, 25% Syrian, 20% Eritrean, 5% Chechen, 5% Iraqi, 5% Kazakh, 5% Pakistani, 5% Palestinian, 5% Somali	Mean: 26.3 (range: 18-41)	M- 90%	NR	NR	NR	Autopsies
Hocking 2015a (40)	All without a history of having been diagnosed with (or treated for) a mental disorder	Community	49% South Asian (Pakistani, Sri Lankan), 27% Zimbabwean, 12% Afghani, 12% Middle Eastern (Iranian, Iraqi, Lebanese)	≥18	M- 70%	NR	59% experienced between 11-26 traumatic events	NR	Hopkins Symptoms Checklist (HSC20)
Hollander 2013 (41)	External causes of death	Community	NR	Range: 18-64	M- 100%	NR	NR	up to 15 years	The Cause of Death Register
Mezey 1960 (56)	Patients with diagnosed psychiatric disorders	Hospital clinic	All Hungarian	65% between 18-40 years old	M- 75%	52% Roman Catholic, 14% Protestant, 13% Jewish, 3% Other	NR	NR	Clinical assessment, hospital records and contacting relatives
Nguyen 1984 (59)	Patients referred for mental health/psychiatric consult	Psychiatric clinic	87% Vietnamese/ Chinese-Vietnamese, 13% other Southeast Asian	Mean: 28 (range: 9-75)	F- 56%	NR	NR	NR	Medical records (referral reasons)

Author, Pub. Year	Selection Criteria/ Study Denominator	Setting	Ethnicity of Displaced People	Age (y)*	Gender	Religion	Trauma Exposure	Duration of Resettlement	Data Collection Source or Measurement
Premand 2018a (65)	Patients attending psychiatric outpatient clinic	Psychiatric clinic in Geneva	37% Balkan, 31% Sub-Saharan African	≥20	F- 51%	NR	NR	NR	Hospital medical records
Refugees in refugee camps or with temporary protection:									
Jahangir 1998 (46)	Outpatients being treated for depression at a psychiatric facility	Refugee camps	All Afghani	NR	NR	100% Muslim	NR	NR	Clinical assessment
Lama 2016 (51)	Patients admitted to a psychiatric hospital	Psychiatric hospital	All Syrian	34.5 (14.0)	M- 54%	NR	NR	NR	Hospital medical records
Yüzbaşıoğlu 2019 (92)	Patients admitted to an emergency department in Ankara	Emergency department	54% Iraqi, 21% Syrian, 9% Afghani	Median: 24 (IQR: 17, 33)	M- 61%	NR	NR	NR	Hospital medical records
Asylum seekers:									
Allodi 1982 (19)	Victims of torture referred to a psychiatrist	Psychiatric clinic	All Latin American	Mean: 26.9 (range: 10-46)	M- 78%	NR	All victims of torture	NR	Clinical assessment
Aronsson 2009 (22)	Children with severe loss of activities of daily living limitation	Multi-professional treatment program	48% Central Asian, 21% Caucasian, 31% other	Mean: 14.4 (range: 7-19)	F- 59%	NR	72% had traumatic experiences according to DSM-IV criteria, and 69% had suffered separation/ loss of close relatives	NR	Clinical assessment
Belz 2017 (23)	Referrals to clinic due to mental distress	Refugee reception center	NR	31.6 (10.6)	M- 66%	NR	97% reported man-made traumatizing events, with the majority reporting multiple traumatizing	NR	Clinical assessment and questionnaires

Author, Pub. Year	Selection Criteria/ Study Denominator	Setting	Ethnicity of Displaced People	Age (y)*	Gender	Religion	Trauma Exposure	Duration of Resettlement	Data Collection Source or Measurement
							events		
Bolton 2014 (27)	Patients diagnosed with depression and/or posttraumatic stress	3 local service organizations	All Burmese	Mean: 35.6 (range: 18-85)	F- 63%	NR	All survivors of imprisonment, torture, and related traumas	Mean: 5.5 years (range: 0-35)	Interview with Hopkins Symptom Checklist 25 (HSCL-25)
Brown 2019 (28)	Intoxication-related emergency department admissions	Emergency department	17% Eritrean, 12% Syrian, 12% Afghani, 11% Somali, 5% Iraqi, 43% other	Median: 29 (IQR: 22, 27)	M- 68%	NR	NR	NR	Hospital medical records
Ferrada-Noli 1996b (34)	Patients with diagnosed PTSD	Psychiatric clinics	27% Iranian, 19% Latin American, 14% Lebanese, 8% Iraqi, 6% Bangladeshi	Mean: 31	M- 63%	55% Muslim, 30% Christian	All traumatised	NR	Clinical assessment (checklist)
Hocking 2015b (40)	All without a history of having been diagnosed with (or treated for) a mental disorder	Community	70% South Asian (Pakistani, Sri Lankan), 12% Zimbabwean, 6% Afghani, 11% Middle Eastern (Iranian, Iraqi, Lebanese)	≥18	M- 89%	NR	61% experienced between 11-26 traumatic events	NR	Hopkins Symptoms Checklist (HSC20)
Hougen 1988 (43)	Half were selected from torture victims (in Denmark)	Community	Lebanese	Range: 18 to 51	M- 100%	NR	50% were selected from torture victims	Range: 4-19 months	Psychological assessment
Neuner 2010 (58)	Patients diagnosed with PTSD and referred for consult	Psychological clinic for refugees	78% Turkish, 13% Balkan, 9% African	31.4 (7.8)	M- 69%	NR	88% had torture experiences; 91% witnessed a violent assault on a familiar person; 72% had been in a war zone	Mean: 56 months	Psychological assessment and structured interviews
Premand 2018b (65)	Patients attending psychiatric outpatient clinic	Psychiatric clinic in	37% Balkan, 31% Sub-Saharan	≥20	F- 51%	NR	NR	Median: 39 months	Hospital medical records

Author, Pub. Year	Selection Criteria/ Study Denominator	Setting	Ethnicity of Displaced People	Age (y)*	Gender	Religion	Trauma Exposure	Duration of Resettlement	Data Collection Source or Measurement
		Geneva	African					(range 6-342)	
Ramel 2015 (67)	Unaccompanied refugee minors admitted to psychiatric unit	Psychiatric unit	75% Afghani	15.9 (1.5)	M- 95%	NR	NR	NR	Retrospective questionnaire to the admitting physicians
Reko 2015 (68)	Patients attending psychiatric emergency service	Mental health centre	35% European, 22% African, 22% Middle Eastern, 13% Asian	32.6 (9.4)	M- 61%	NR	NR	Mean 19 months	Medical charts assessing suicidal risk according to a standardized instrument
Richter 2018a (69)	Help-seekers of psychiatric services in Bavaria	Admission center for asylum seekers	28% Russian, 24% Afghani, 23% Iranian, 10% Iraqi, 6% Azerbaijani	32.2 (11.7)	F- 52%	50% Muslim, 22% Christian, 26% other	NR	NR	Clinical assessment using Montgomery Asberg Depression Rating Scale (MADRS) item 10
Richter 2018b (69)	Sample excluding help-seekers of psychiatric services in Bavaria	Admission center for asylum seekers	39% Iranian, 23% Iraqi, 15% Afghani, 9% Russian, 5% Azerbaijani	31.7 (9.1)	F- 34%	59% Muslim, 18% Christian, 21% other	NR	NR	Clinical assessment using Montgomery Asberg Depression Rating Scale (MADRS) item 10
Schoretsanitis 2018 (74)	Psychiatric emergency department patients	Emergency department	28% Sub-Saharan African, 23% Middle Eastern, 22% Northwestern African, 18% Central-South Asian, 9% Eastern European	29.9 (9.13)	M- 75%	NR	NR	NR	Hospital medical records
Mixed samples of refugees & asylum seekers:									
Betancourt 2017 (24)	Children/adolescents referred for trauma-related mental health services	56 centres providing youth mental health services	35% Latin American, 26% African, 21% European, 12% Asian	13.07 (4.13)	M- 52%	NR	All trauma-exposed. Mean number of trauma types (SD): 5.43 (2.46)	NR	Core Data Set quality improvement initiative conducted by trained clinicians. Used multiple sources to complete the assessment (directly from clients and caregivers, collateral reports and

Author, Pub. Year	Selection Criteria/ Study Denominator	Setting	Ethnicity of Displaced People	Age (y)*	Gender	Religion	Trauma Exposure	Duration of Resettlement	Data Collection Source or Measurement
									records)
Lerner 2016 (53)	Survivors of torture	A torture survivor's program of services	36% West African, 24% Asian, 15% Eastern European, 10% Central African, 14% other African	34 (9.5)	M- 67%	40% Muslim, 35% Christian	All survivors of torture	NR	Clinical intake records of a semi-structured intake questionnaire
Internally displaced people:									
Olema 2014 (63)	Adolescents in Northern Uganda; half were selected from those who were formerly abducted by the rebel army	Internally displaced people camps	All Ugandan	14.6 (1.5)	NR	NR	Half had been formerly abducted. Among those not abducted, mean number of war trauma event types was 7.1 (SD 4.2)	NR	Mini International Neuropsychiatric Interview

*Reported as mean (SD) unless otherwise specified.

Abbreviations: IQR=inter-quartile range; NR=not reported; PTSD=post-traumatic stress disorder; y=year.
