# Supplementary material for: Suicide rates and suicidal behaviour in displaced people: A systematic review
Source: PLoS One. 2022 Mar 10;17(3):e0263797. doi: 10.1371/journal.pone.0263797 (PMC8912254; doi:10.1371/journal.pone.0263797)
Supplement: S3 Table — (PDF) [file pone.0263797.s003.pdf]

S3 Table. Suicide rates per 100,000 person-years using specific samples.

Author, Publication Year	Study Denominator	Population Type	Host Country	Data Source	Study Dates	Suicides, n out of N	Suicide Rate, per 100,000 person-years
Gleich 2018 (36)	Forensic autopsies of deceased refugees ordered by the prosecuting attorney's office in Munich	Refugees granted asylum	Germany	Autopsies	2014-2015	2 out of 20	not estimable
Hollander 2013 (41)	External causes of death	Refugees granted asylum	Sweden	Vital events registration	1998-2006	~ 40% of the external causes of death (n and N not reported)	not estimable
