# Supplementary material for: Suicide rates and suicidal behaviour in displaced people: A systematic review
Source: PLoS One. 2022 Mar 10;17(3):e0263797. doi: 10.1371/journal.pone.0263797 (PMC8912254; doi:10.1371/journal.pone.0263797)
Supplement: S4 Table — (PDF) [file pone.0263797.s004.pdf]

S4 Table. Suicide attempt prevalence percentage using specific samples, ordered by population type (and then by author).

Author, Pub. Year	Study Denominator	Population Type	Host Country	Data Source	Study Dates	Suicide Attempts, n out of N	Time-frame*	Prevalence, % (95% CI) †
Ferrada-Noli 1996a (34)	Patients with diagnosed PTSD	Refugees granted asylum	Sweden	Cross-sectional	NR	11 out of 32	NR	34.4 (18.6 to 53.2)
Mezey 1960 (56)	Patients with diagnosed psychiatric disorders	Refugees granted asylum	United Kingdom	Cross-sectional	1957-1959	9 out of 82	presenting symptom	11.0 (5.1 to 19.8)
Nguyen 1984 (59)	Patients referred for mental health/psychiatric consult	Refugees granted asylum	Canada	Cross-sectional	1978-1983	29 out of 118	presenting symptom	24.6 (17.1 to 33.4)
Jahangir 1998 (46)	Patients treated for depression	Refugees in camps	Pakistan	Cross-sectional	1979-1991	4 out of 117	lifetime	3.4 (0.94 to 8.5)
Lama 2016 (51)	Patients admitted to a psychiatric hospital	Refugees w/ temporary protection	Lebanon	Cross-sectional	2011-2013	15 out of 106	current state	14.2 (8.1 to 22.3)
Yüzbaşıoğlu 2019 (92)	Patients admitted to emergency department	Refugees w/ temporary protection	Turkey	Cross-sectional	2016-2018	32 out of 310	current state	10.3‡ (7.2 to 14.3)
Allodi 1982 (19)	Victims of torture referred to a psychiatrist	Asylum seekers	Canada	Cross-sectional	1977-1979	4 out of 41	since torture	9.8 (2.7 to 23.1)
Aronsson 2009 (22)	Children with severe loss of activities of daily living limitation	Asylum seekers	Sweden	Specific monitoring	NR	12 out of 29	NR	41.4 (23.5 to 61.1)
Ferrada-Noli 1996b (34)	Patients with diagnosed PTSD	Asylum seekers	Sweden	Cross-sectional	NR	8 out of 32	NR	25.0 (11.5 to 43.4)
Reko 2015 (68)	Patients attending psychiatric emergency service	Asylum seekers	Denmark	Cross-sectional	2013	5 out of 24	current state	20.8 (7.1 to 42.2)
Schoretsan-itis 2018 (74)	Psychiatric emergency department patients	Asylum seekers	Switzer-land	Cross-sectional	2012-2017	3 out of 119	current presentation	2.5 (0.52 to 7.2)

*Timeframe refers to either how far back participants were asked about the occurrence of a suicide attempt (for example, in the past year), or the follow-up time.

†Confidence intervals (of prevalence percentages) were calculated using Stata 12.

‡This study found a prevalence in the host population of 8.2% (1247/15,219).

Abbreviations: NR=not reported; PTSD=post-traumatic stress disorder; Pub.=Publication; w/=with.
