# Supplementary material for: Suicide rates and suicidal behaviour in displaced people: A systematic review
Source: PLoS One. 2022 Mar 10;17(3):e0263797. doi: 10.1371/journal.pone.0263797 (PMC8912254; doi:10.1371/journal.pone.0263797)
Supplement: S5 Table — (PDF) [file pone.0263797.s005.pdf]

S5 Table. Suicidal ideation prevalence percentage using specific samples*, ordered by population type (and then by author).

Author, Pub. Year	Study Denominator	Population Type	Host Country	Data Source	Study Dates	Suicidal Ideation, n out of N	Time-frame†	Prevalence, % (95% CI)‡
Ferrada-Noli 1996a (34)	Patients with diagnosed PTSD	Refugees granted asylum	Sweden	Cross-sectional	NR	10 out of 32	NR	31.3 (16.1 to 50.0)
Hocking 2015a (40)	All without a history of having been diagnosed with (or treated for) a mental disorder	Refugees granted asylum	Australia	Cross-sectional	NR	1 out of 33	current state	3.0 (0.07 to 15.8)
Premand 2018a (65)	Patients attending psychiatric outpatient clinic	Refugees granted asylum	Switzer-land	Cross-sectional	2012	10 out of 36	14 months §	27.8¶ (14.2 to 45.2)
Jahangir 1998 (47)	Patients treated for depression	Refugees in camps	Pakistan	Cross-sectional	1979-1991	16 out of 117	lifetime	13.7 (8.0 to 21.3)
Lama 2016 (51)	Patients admitted to a psychiatric hospital	Refugees w/ temporary protection	Lebanon	Cross-sectional	2011-2013	26 out of 106	current state	24.5 (16.7 to 33.8)
Belz 2017 (23)	Referrals to clinic due to mental distress	Asylum seekers	Germany	Cross-sectional	2012-2015	46 out of 85	acute or latent	54.1 (43.0 to 65.0)
Bolton 2014 (27)	Patients diagnosed with depression and/or posttraumatic stress	Asylum seekers	Thailand	Baseline RCT data	2011	20 out of 347	current state	5.8 (3.6 to 8.8)
Brown 2019 (28)	Intoxication-related emergency department admissions	Asylum seekers	Switzer-land	Cross-sectional	2013-2016	10 out of 92	NR	10.9# (5.3 to 19.1)
Ferrada-Noli 1996b (34)	Patients with diagnosed PTSD	Asylum seekers	Sweden	Cross-sectional	NR	7 out of 32	NR	21.9 (9.3 to 40.0)
Hocking 2015b (40)	All without a history of having been diagnosed with (or treated for) a mental disorder	Asylum seekers	Australia	Cross-sectional	NR	16 out of 95	current state	16.8 (9.9 to 25.9)
Premand 2018b (65)	Patients attending psychiatric outpatient clinic	Asylum seekers	Switzer-land	Cross-sectional	2012	30 out of 83	14 months §	36.2¶ (25.9 to 47.4)
Reko 2015 (68)	Patients attending psychiatric emergency service	Asylum seekers	Denmark	Cross-sectional	2013	18 out of 24	current state	75.0 (53.3 to 90.2)
Richter 2018a (69)	Help-seekers of psychiatric services	Asylum seekers	Germany	Cross-sectional	2011-2012	41 out of 158	current state	26.0 (19.3 to 33.5)
Richter 2018b (69)	Sample not including help-seekers of psychiatric services	Asylum seekers	Germany	Cross-sectional	2011-2012	8 out of 125	current state	6.4 (2.8 to 12.2)
Schoretsan-itis 2018 (74)	Psychiatric emergency department patients	Asylum seekers	Switzer-land	Cross-sectional	2012-2017	30 out of 119	current present ation	25.2 (17.7 to 34.0)
Lerner 2016 (53)	Survivors of torture	Mixed refugee and asylum seeker populations	USA	Cross-sectional	2010-2013	78 out of 267	current state	29.2 (23.8 to 35.1)
Olema 2014 (63)	Half were selected from those formerly abducted	Internally displaced	Uganda	Cross-sectional	2006	10 out of 100	current state	10.0 (4.9 to 17.6)

*In addition to the studies above, two reported only on a composite of suicidal ideation or suicide attempt, as follows: (i) Hougen 1988 (43) (asylum seekers in Denmark) found it to be 29.2% (7/24); and (ii) Neuner 2010 (58) (asylum seekers in Germany) found 40.6% (13/32). Also, a 3rd study by

Ramel 2015 (67) (asylum seekers in Sweden) reported on a composite of self-harm or suicidal behaviour and estimated a prevalence of 76% (42/56), which was higher than in the host population (58% or 119/205). A 4th study by Betancourt 2017 (24) (mixed refugees and asylum seekers in USA) reported on "suicidality judged as probable/ definite pathology" (undefined) and found a prevalence of 5.4% (3/56), which was lower than the host population's prevalence of 9.3% (12/129; propensity score matched).

[†]Timeframe refers to either how far back participants were asked about the occurrence of a suicide attempt (for example, in the past year), or the follow-up time.

[‡]Confidence intervals (of prevalence percentages) were calculated using Stata 12.

[§]Median 14 months, range 3-92.

[¶]This study found a prevalence in the host population of 30.8% (37/120).

[#]This study found a prevalence in the host population of 12.2% (225/1841).

Abbreviations: NR=not reported; PTSD=post-traumatic stress disorder; Pub.=Publication; RCT=randomised controlled trial; w/=with.
